# Envelope Deglycosylation Enhances Antigenicity of HIV-1 gp41 Epitopes for Both Broad Neutralizing Antibodies and Their Unmutated Ancestor Antibodies

**DOI:** 10.1371/journal.ppat.1002200

**Published:** 2011-09-01

**Authors:** Ben-Jiang Ma, S. Munir Alam, Eden P. Go, Xiaozhi Lu, Heather Desaire, Georgia D. Tomaras, Cindy Bowman, Laura L. Sutherland, Richard M. Scearce, Sampa Santra, Norman L. Letvin, Thomas B. Kepler, Hua-Xin Liao, Barton F. Haynes

**Affiliations:** 1 Duke Human Vaccine Institute, Duke University School of Medicine, Durham, North Carolina, United States of America; 2 Department of Medicine, Duke University School of Medicine, Durham, North Carolina, United States of America; 3 Department of Chemistry, University of Kansas, Lawrence, Kansas, United States of America; 4 Department of Immunology, Duke University School of Medicine, Durham, North Carolina, United States of America; 5 Deparment of Surgery, Duke University School of Medicine, Durham, North Carolina, United States of America; 6 Center for Computational Immunology, Duke University School of Medicine, Durham, North Carolina, United States of America; 7 Beth Israel Deaconess Medical Center, Harvard Medical School, Boston, Massachusetts, United States of America; University of Zurich, Switzerland

## Abstract

The HIV-1 gp41 envelope (Env) membrane proximal external region (MPER) is an important vaccine target that in rare subjects can elicit neutralizing antibodies. One mechanism proposed for rarity of MPER neutralizing antibody generation is lack of reverted unmutated ancestor (putative naive B cell receptor) antibody reactivity with HIV-1 envelope. We have studied the effect of partial deglycosylation under non-denaturing (native) conditions on gp140 Env antigenicity for MPER neutralizing antibodies and their reverted unmutated ancestor antibodies. We found that native deglycosylation of clade B JRFL gp140 as well as group M consensus gp140 Env CON-S selectively increased the reactivity of Env with the broad neutralizing human mAbs, 2F5 and 4E10. Whereas fully glycosylated gp140 Env either did not bind (JRFL), or weakly bound (CON-S), 2F5 and 4E10 reverted unmutated ancestors, natively deglycosylated JRFL and CON-S gp140 Envs did bind well to these putative mimics of naive B cell receptors. These data predict that partially deglycoslated Env would bind better than fully glycosylated Env to gp41-specific naïve B cells with improved immunogenicity. In this regard, immunization of rhesus macaques demonstrated enhanced immunogenicity of the 2F5 MPER epitope on deglyosylated JRFL gp140 compared to glycosylated JRFL gp140. Thus, the lack of 2F5 and 4E10 reverted unmutated ancestor binding to gp140 Env may not always be due to lack of unmutated ancestor antibody reactivity with gp41 peptide epitopes, but rather, may be due to glycan interference of binding of unmutated ancestor antibodies of broad neutralizing mAb to Env gp41.

## Author Summary

Critical to the design of an effective HIV-1 vaccine that will induce long-lasting broadly neutralizing antibodies is to understand why broad neutralizing antibodies are not induced. One hypothesis is that there are “holes” in the naïve B cell repertoires for unmutated B cell receptors that can bind to HIV-1 envelope (Env) neutralizing epitopes. In this paper, we test this hypothesis for the rare HIV-1 envelope gp41 broad neutralizing monoclonal antibodes (mAbs), called 2F5 and 4E10, and show that indeed, fully glycosylated Env does not bind to inferred unmutated ancestor antibodies (mimics of naïve B cell receptors) of mAbs 2F5 and 4E10, but that partially deglycosylated Envs that have had glycans removed under non-denaturing conditions, did bind to 2F5 and 4E10 unmutated ancestor antibodies. Thus, rather than there being a lack of existence of germline B cell receptors for gp41 broad neutralizing antibodies, one impediment to induction of gp41 broad neutralizing antibodies may be glycan interference with unmutated antibody binding to gp41 envelope.

## Introduction

Two rare human monoclonal antibodes (mAbs), 2F5 and 4E10, bind to linear epitopes in the gp160 membrane proximal external region (MPER) [1], [2]. The core sequence of 2F5 epitope is aa 662–668 (ELDKWAS), and that of 4E10 is aa 671–676 NWFDIT [3]. The crystal structures of the 2F5 and 4E10 Fabs in complex with peptides containing their gp41 core epitopes demonstrated that only a relatively small portion of the CDRH3 antibody regions bound the MPER [4], [5]. Both mAbs 2F5 and 4E10 are polyreactive for lipids and have long hydrophobic heavy chain complementarity determining regions (HCDR3s) [6]. Hydrophobic HCDR3 loops of the variable region of the heavy chain (VH) of both mAbs 2F5 and 4E10 bind viron lipids in a two-step conformational change model that is required for antibody neutralization [7]–[11]. Thus, mAbs 2F5 and 4E10 use their autoreactive specificities to mediate anti-HIV-1 activity [8], [12]. The autoreactivity of mAbs 2F5 and 4E10 has raised the hypothesis that their rarity is due to tolerance control due to their polyreactivity with host antigens [6]. Indeed, the creation of knock-in mice with 2F5 [13] and 4E10 [14] VHs have demonstrated this to be the case in these mice. MAbs 2F5 and 4E10 rarely bind well to gp140 oligomers, possibly in part due to lack of formation of the gp41 intermediate form that binds to mAbs 2F5 and 4E10 [15]. Xiao *et al*. [16] have suggested that an additional reason for the rarity of broadly neutralizing antibodies in general, and 2F5-like antibodies in particular, is the lack of reactivity of HIV-1 Env with the unmutated ancestors of broad neutralizing antibodies. That is, this hypothesis states that there are “holes” in the naïve B cell repertoire resulting in lack of unmutated antibodies that can bind and respond to conserved Env broadly neutralizing epitopes.

Glycosylation can modulate the binding of antibodies to gp120 with gain or loss of a glycosylation site in a virus mutant affecting both Env antigenicity and virus neutralization sensitivity [17]–[23]. Analysis of HIV-1 Env mutated by site-directed mutagenesis has indicated that Env glycans in the first and second variable (V1/V2) loops [24], the third (V3) [25] and fourth variable (V4) loops of gp120 [26] modulate the binding of antibodies to Env. Yuste *et al*. demonstrated that glycosylation site deletion mutants of SIVmac239 induced neutralizing antibodies to a novel conserved region C-terminal to the gp41 immunodominant region [23]. In addition, two recent studies have demonstrated that the N-linked glycosylation site at Env aa 413 is associated with induction of broad neutralizing antibodies [27], [28].

In this study, we have partially deglycosylated JRFL gp140 and group M consensus gp140 (CON-S) [29] Env proteins under native, non-denaturing conditions, and demonstrated enhanced binding of mAbs 4E10 and 2F5 to deglycosylated Env. While the reverted unmutated ancestor antibodies of 2F5 and 4E10 were either non-reactive (with glycosylated JRFL Env), or poorly reactive (with glycosylated CON-S Env), they reacted well with partially deglycosylated gp140 HIV-1 envelope, indicating for some HIV-1 envelopes, an intact naïve B cell repertoire for 2F5 and 4E10 gp41 neutralizing epitopes.

## Results

### Analysis of Deglycosylated HIV-1 gp140 Env Proteins in SDS-PAGE, Western Blot Analysis and ELISA

**Figure 1:**
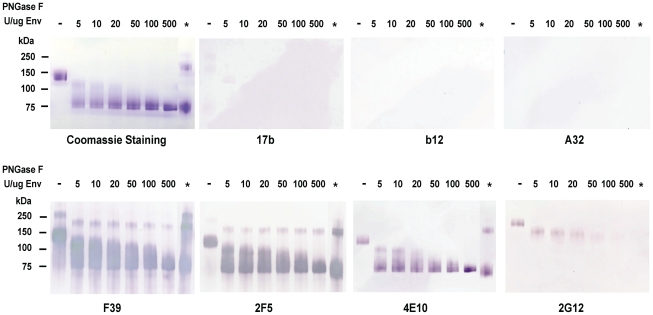
Analysis of deglycosylated JRFL protein in reducing SDS-PAGE and Western-blotting with a panel of HIV-1 MAbs. WT glycosylated and deglycosylated JRFL gp140 Env proteins were fractionated in 4–12% SDS-PAGE under reducing conditions, stained with coommassie blue or were transferred to nitrocelose membranes and blotted with a panel of human anti-HIV-1 mAbs as indicated. WT glycosylated JRFL gp140 Env protein is labeleled as [-] on the top of the lane. The amount of PNGase F (in U) used in the reaction per µg of protein is indicated on the top of lanes with progressively deglycosylation. Lane * refers to treatment of denatured JRFL gp140 protein with PNGaseF (500 U) at 37 degrees for 2 hours. Progressive reductions in molecular weight of JRFL gp140 with increasing amounts of PNGase used to deglycosylate JRFL gp140 Env are shown in Coomassie staining gels and Western blots using mAbs F39, 2F5 and 4E10. The higher molecular weight band(s) shown in lane * in the coomassie stained gel and in Western blots by F39, 2F5 and 2G12 were due to the incomplete reduction of JRFL gp140 Env protein (see coomassie stained gel in Figure S1).

To deglycosylate maximally HIV-1 Env under non-denaturing conditions, while in order to maintaining the native conformations, HIV-1 JRFL recombinant gp140 Env was treated with a wide dose range of PNGase F. Figure 1 shows JRFL gp140 Env protein treated with progressive deglycosylation (PNGase F 5, 10, 20, 50, 100 and 500 U per µg of Env protein) analyzed in SDS-PAGE and Western blots. After deglycosylation, the molecular mass of HIV-1 JRFL gp140 Env protein was progressively reduced with increasing doses of PNGase F, with maximal deglycosylation under non-denaturing conditions using 500 U PNGase F per 1 µg of Env. At this dose, JRFL Env gp140 molecular mass was reduced to near that of Env treated with PNGase F under denaturing conditions (approximately 80 kDa Env shown in lanes with an asterisk in Figure 1). In blue BN-PAGE gel, analysis, JRFL gp140 Env deglycosylated under native conditions using 500U/ug Env PNGase F migrated as monomers, dimers and trimers in the same manner as did wild-type (WT) glycosylated JRFL Env gp140 (Figure S1A).

In SDS-PAGE and Western blot analysis under reducing conditions, there was weak or no binding of deglycosylated JRFL Env gp140 to the co-receptor binding site mAb 17b [30], to the CD4 binding site mAb 1b12 [20], or to the gp120 conformational-dependent mAb, A32 [31] (Figure 1). However, the binding of anti-V3 loop mAb F39F [31] to JRFL gp140 was not affected by deglycosylation when assayed under these conditions (Figure 1). In contrast, the binding of anti-carbohydrate mAb 2G12 to deglycosylated JRFL gp140 was decreased with progressive native deglycosylation. The binding of gp41 MPER human mAb 2F5 was not inhibited by deglycosylation or reduction, while progressive deglycosylation appeared to increase binding of the gp41 MPER antibody 4E10 to HIV-1 JRFL gp140 protein. Maximal binding of mAb 4E10 to JRFL Env protein was seen by using the deglycosylated Env treated with the highest level of PNGase F (500 U per µg of Env). This enhanced level of 4E10 binding was similar to the level of 4E10 binding to fully PNGase F-deglycosylated Env under denaturing conditions (asterisk, Figure 1).

**Figure 2:**
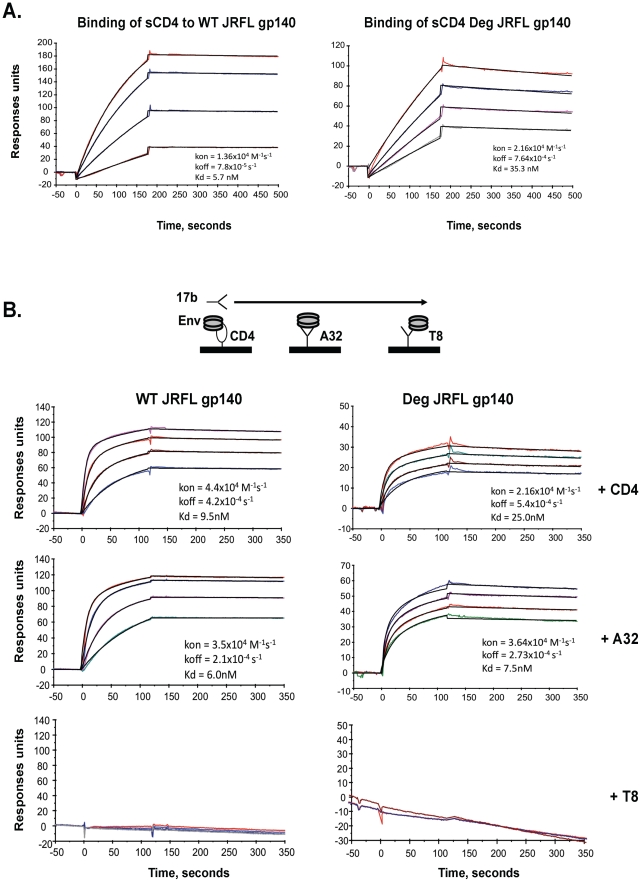
Analysis of antigenic epitopes expressed on WT glycosylated and deglycosylated JRFL gp140 by surface plasmon resonance (SRP). SPR assays were performed as described in Materials and Methods. Shown are the ability of WT glycosylated and deglycosylated JRFL gp140 to bind to sCD4 (Panel A) and to mAb 17B (Panel B). sCD4 or HIV-1 mAbs T8 and A32 were covalently immobilized to a CM5 sensor chip (BIAcore), and WT glycosylated (10 to 75 µg/ml) and deglycosylated JRFL gp140 were injected over each surface (100 and 300 ug/ml, respectively). To determine induction of 17b mAb binding to WT glycosylated and deglycosylated JRFL gp140, Env proteins were captured on individual flow cells immobilized with sCD4 (80–120 response units or RU) or mAb A32 (∼130–300RU) or T8 (∼340–450 RU), with about the same RU of Env gp140 captured on each surface prior to injection of varying concentrations of 17 mAb. Following stabilization of each of the surfaces, mAb 17b (10–100 µg/ml) was injected and flowed over each of the Env captured surfaces as illustrated in the diagram above the SPR profile in Panel B. Each analysis was performed at least twice.

To compare antigenic and functional epitopes, glycosylated and natively deglycosylated JRFL Envs were assayed for their ability to bind sCD4, and for their ability to undergo CD4 and mAb A32 induction of the CCR5 binding site as defined by binding of mAb 17b to Env. Thus, sCD4, mAb A32, or gp120 C1 control mAb T8 was immobilized on a surface plasmon reasonance (SPR) sensor chip and then either WT glycosylated or deglycosylated JRFL gp140 were flowed over the chip, followed by flowing over mAb 17b (Figures 2A, 2B). First, we found that WT glycosylated JRFL gp140 Env bound to sCD4 with Kd of 5.7 nM, while deglycosylated JRFL gp140 protein bound sCD4 with Kd of 35.3 nM (Figure 2A). The WT glycosylated JRFL gp140 Env was induced to bind mAb17b following binding to either sCD4 or mAb A32 with Kd of 9.5 nM and 6.0 nM, respectively, while the deglycosylated JRFL gp140 Env was induced to bind mAb17b following binding to either sCD4 or MAb A32 with slightly higher Kd of 25.0 nM and 7.5 nM, respectively. No binding of mAb 17b binding was observed following the binding of either WT glycosylated or native deglycosylated Env to gp120 C1 mAb T8 (Figure 2B). Thus, native deglycosylation of JRFL gp140 Env did not perturb the ability of the Env to undergo sCD4 and mAb A32-induced exposure of the CCR5 Env binding site.

### Increased Affinity of 4E10 and 2F5 mAbs for Deglycosylated Env

**Figure 3:**
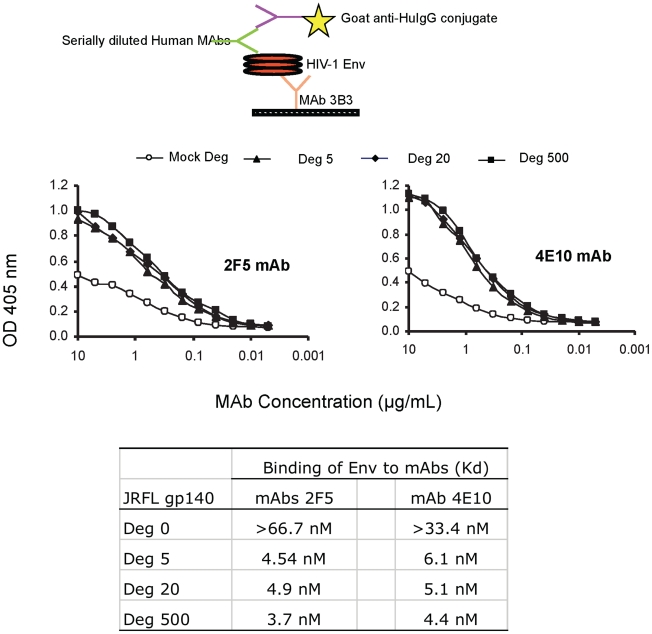
Binding kinetics of mAbs 2F5 and 4E10 to WT glycosylated and deglycosylated JRFL gp140. Binding of mAbs 2F5 and 4E10 to WT glycosylated and progressively deglycosylated JRFL gp140 proteins was determined in direct ELISA. An anti-HIV-1 Env C1 region antibody 3B3 [31] was coated on the 96-well plates to capture glycosylated and progressively deglycosylated JRFL gp140 proteins following by incubation with mAbs 2F5 and 4E10. The mAbs 2F5 and 4E10 were assayed at the concentrations as indicated on the x-axis. Data presented are representative of 3 independent experiments.

To ask if indeed, the deglycosylation of JRFL oligomer induced exposure of the MPER as suggested by reduced SDS-PAGE Western blot analysis, we performed indirect ELISAs using serially diluted human mAbs and a fixed amount of Env proteins captured on ELISA plates by anti-gp120 C1 mouse mAb 3B3 [32]. In this assay, both mAbs 2F5 and 4E10 bound glycosylated JRFL Env protein with the apparent Kd of approximately 67 nM and 33 nM, respectively, while both mAbs 2F5 and 4E10 bound significantly better to the natively deglycosylated JRFL Env with apparent Kds of 4.9 nM and 6.1 nM, respectively (Figure 3). The apparent Kds of mAbs 2F5 and 4E10 binding to natively deglycosylated JRFL Env proteins treated with either 500 U, 20 U or 5 U per µg Env were all similar with apparent Kds of 3.7 nM, 4.9 nM and 4.5 nM for mAb 2F5, and 4.4 nM, 5.1 nM and 6.1 nM for mAb 4E10 at these PNGase F concentrations, respectively (Figure 3).

**Figure 4:**
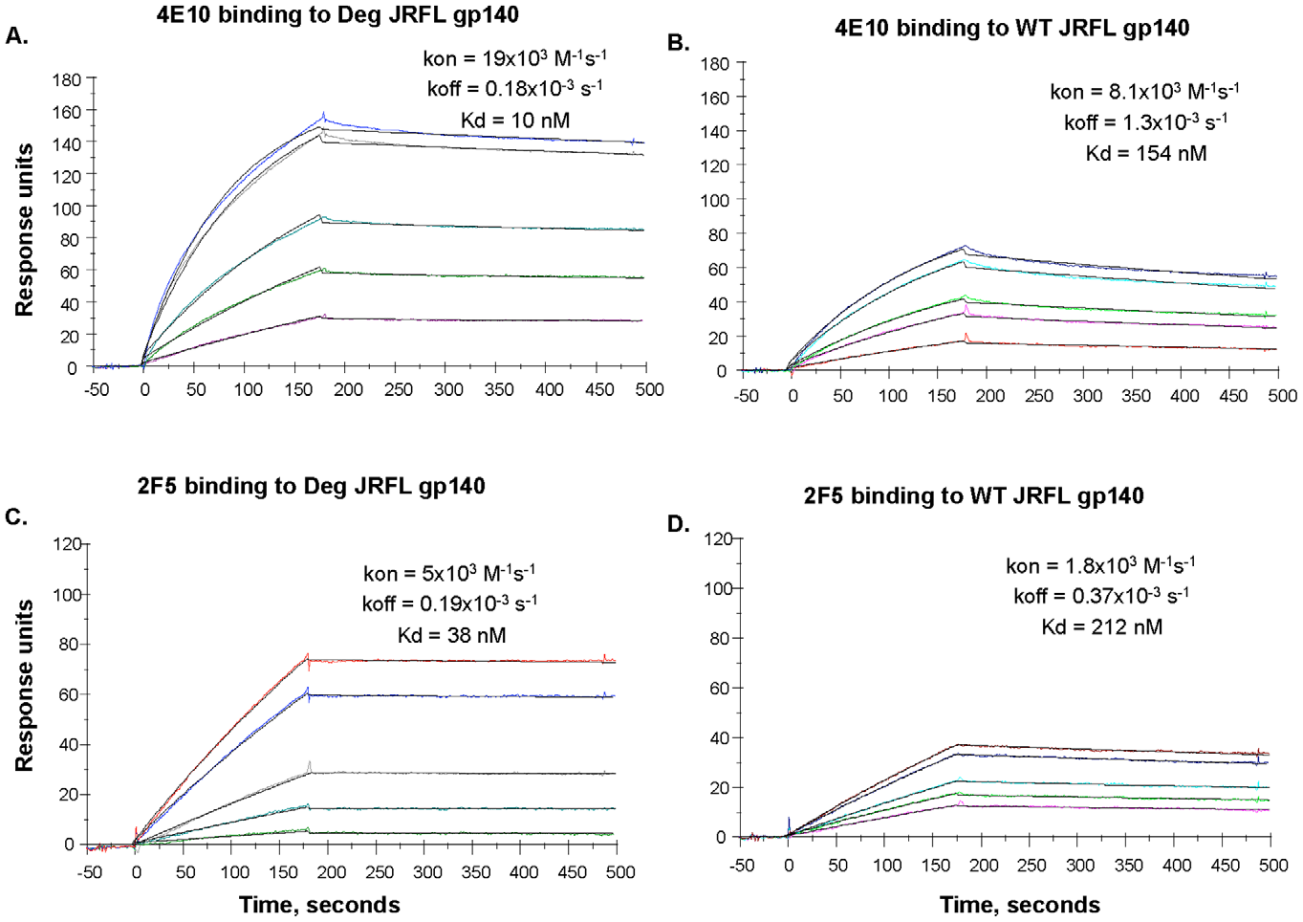
SPR assays for native and deglycosylated JRFL gp140 binding to mAbs 4E10 and 2F5. MAbs 4E10 (Panel A, B) and 2F5 (Panel C, D) were bound to anti-human Ig Fc immobilized on the CM5 sensor chip. Varying concentrations (ranging from 5 to 100 ug/mL) of native (WT) and deglycosylated (Deg) JRFL gp140 Envs were injected over the mAbs on the chip and binding kinetics were recorded. Rate constants and Kd measurements were made using the 1∶1 Langmuir model and data recorded are indicated in the individual panels. Deglycosylation of JRFL gp140 enhanced the Kd of 4E10 and 2F5 binding by ∼15-fold and ∼6-fold respectively. Data shown are representative of two experiments.

We next performed SPR analysis of binding of mAbs 2F5 and 4E10 to WT glycosylated and deglycosylated JRFL Env proteins by flowing either glycosylated or deglycosylated JRFL Env proteins over mAbs 4E10 or 2F5 captured on an SPR sensor chip (Figure 4). Marked enhancement of mAb 4E10 binding to deglycosylated JRFL Env (Figure 4A) was observed compared to the binding of mAb 4E10 to the WT glycosylated JRFL (Figure 4B). SPR analysis demonstrated that the enhancement of 4E10 binding to deglycosylated JRFL proteins was due to both a faster on- rate (K_on_) and a slower off rate (K_off_) that resulted in a net 15-fold decrease in the overall dissociation constant (Kd) of 4E10 binding from 154 nM for glycosylated JRFL Env to 10 nM for native deglycosylated JRFL Env (Figures 4B and 4A). Similar but weaker enhancement (6-fold) of 2F5 mAb binding to deglycosylated JRFL Env was seen in SPR analysis (Kd = 38 nM for 2F5 binding to deglycosylated Env vs. Kd = 212 nM of 2F5 binding to WT glycosylated Env) (Figures 4C and 4D).

### Preserved DC-SIGN Binding By Natively Deglycosylated JRFL Env

**Figure 5:**
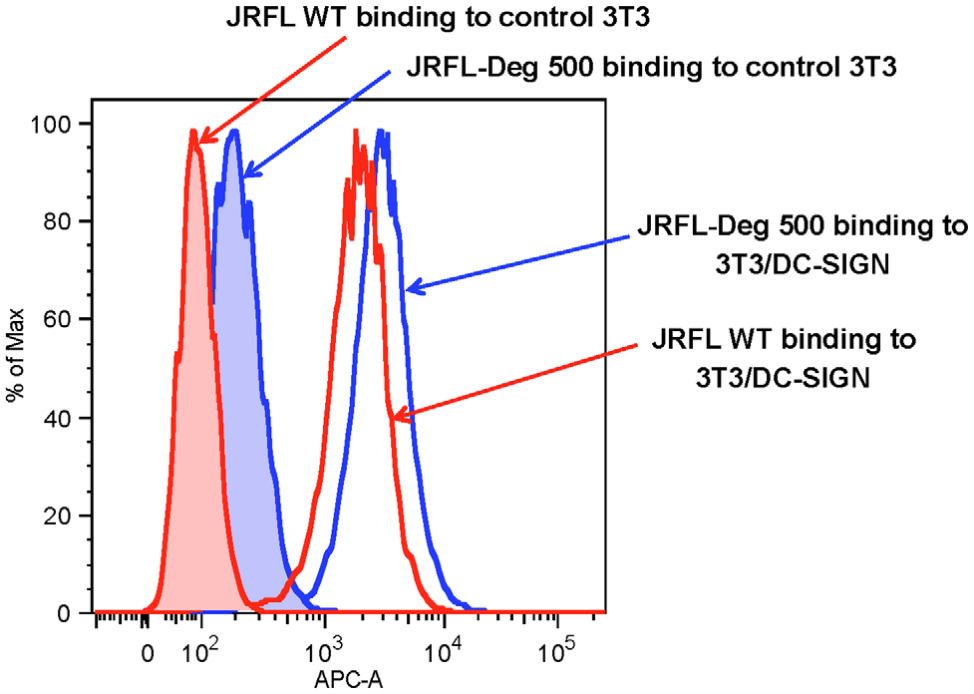
Binding of WT glycosylated and deglycosylated JRFL gp140 Env on DC-SIGN-expressing NIH 3T3 cells. The binding of AF647-labeled WT glycosylated (histogram in red lines and deglycosylated JRFL Env gp140 (histograms in blue lines) to parent NIH 3T3 (histograms shaded) and DC-SIGN-expressing NIH 3T3 cells (histograms not shaded) were analyzed by flow cytometry. Data shown are representative of three experiments.

We next evaluated the binding of natively partially deglycosylated Env to DC-SIGN using AF647-labeled JRFL Env. We found that neither partial deglycosylation nor labeling of AF647 had any effect on the binding of JRFL gp140 Env protein to CD4 expressing TZM-b1 cells (not shown). Natively deglycosylated JRFL gp140 Env (treated with 500 U PNGase F per µg of Env) retained the ability to bind to DC-SIGN-transfected 3T3 cells but not control 3T3 cells (Figure 5) indicating preserved high mannose residues on the PNGase-treated natively partially deglycosylated Env.

### The 2G12 Epitope was Maintained on JRFL Env After Deglycosylation Under Native Conditions

It has previously been reported that binding of mAb 2G12 requires gp120 Env N-linked glycan at positions N286, N323, and N330 [33]. Since native deglycosylation with PNGase F is not complete, and the maximally deglycosylated JRFL gp140 Env (treated with 500 U PNGase F per µg of Env) bound to DC-SIGN transfected 293T cells, we asked if 500 U PNGase F-deglycosylated gp140 could also bind the anti-gp120 carbohydrate-dependent mAb 2G12.

**Figure 6:**
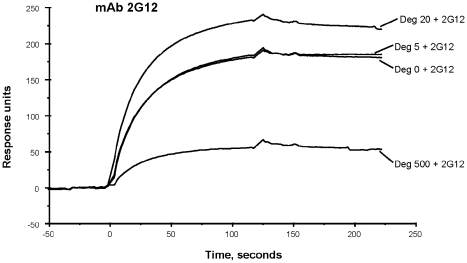
SPR assays for binding of native and deglycosylated JRFL gp140 to mAb 2G12. MAb 2G12 was immobilized on the CM5 chip. The saturated amounts of native and representative deglycosylated proteins were then flown over the mAbs. The binding kinetics were recorded at 25°C. Deglycosylation of JRFLgp140 using lower dose of PGNase (20 U: µg) enhanced the binding of 2G12 but substantially decreased binding of 2G12. Data show binding curves of each deglycosylated and WT glycosylated protein injected at 50 µg/mL and 200 µg/mL respectively, and are representative of two experiments.

Although the binding of 2G12 to deglycosylated JRFL protein was weak in reducing SDS-PAGE Western blot analysis (Figure 1), in SPR analysis in which Env is flowed over captured mAb, binding of 2G12 to JRFL gp140 protein was maintained when Env was treated with PNGase F (Figure 6). While 5U and 20U of PNGase F did not decrease mAb 2G12 binding, 500U of PNGase F treatment did decrease, but did not completely abrogate, mAb 2G12 binding (Figure 6).

### Characterization of Env Carbohydrates Remaining on JRFL gp140 After Deglycosylation Under Non-denaturing Conditions

PNGase F 500 U-treated JRFL gp140 was subjected to enzymatic digestion and LC-MS/MS analysis to define sites and forms of glycan remaining on optimally deglycosylated gp140 (500 U PNGase F) [34], [35]. JRFL gp140 has 27 potential N-linked glycosylation sites, and the occupied sites are composed of high mannose, hybrid, and complex glycans [34], [35]. The remaining glycosylation on these sites was identified using a combination of high resolution MS and MS/MS data of the endoglycosidase-treated glycopeptides, as described in the section od Materials and Methods. The resulting MS data is summarized in Supplemental Table 1. Each peptide containing a potential glycosylation site was detected as either a glycopeptide, a deglycosylated peptide, or as a natively nonglycosylated speices.

**Figure 7:**
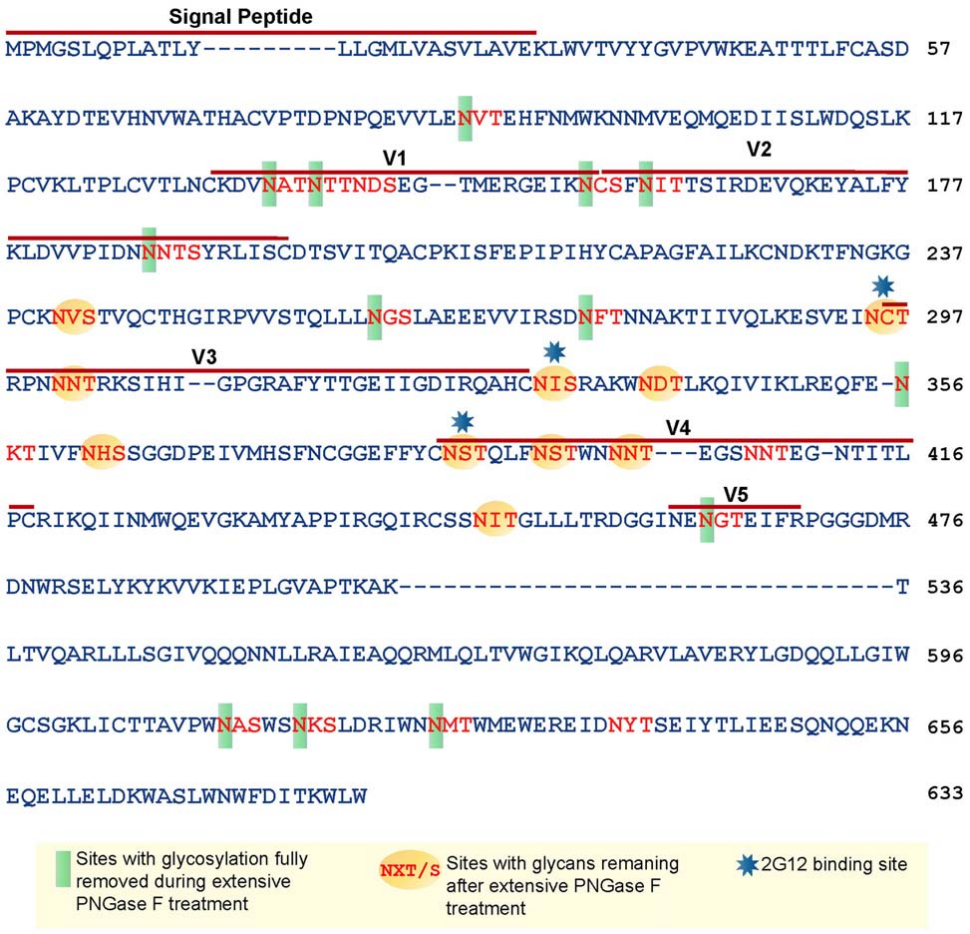
Glycan analysis of native partially deglycosylated JRFL gp140 protein. Native deglycosylation of JRFL gp140 protein was performed using PNGase F (500 unit/µg protein) as described in the materials and methods. Amino acid sequence numbers were based on the HXB2 sequence. The signal peptide and variable regions of the sequence were identified above the sequence. See Table S1 for mass spectrometry analysis data summaries.

**Figure 8:**
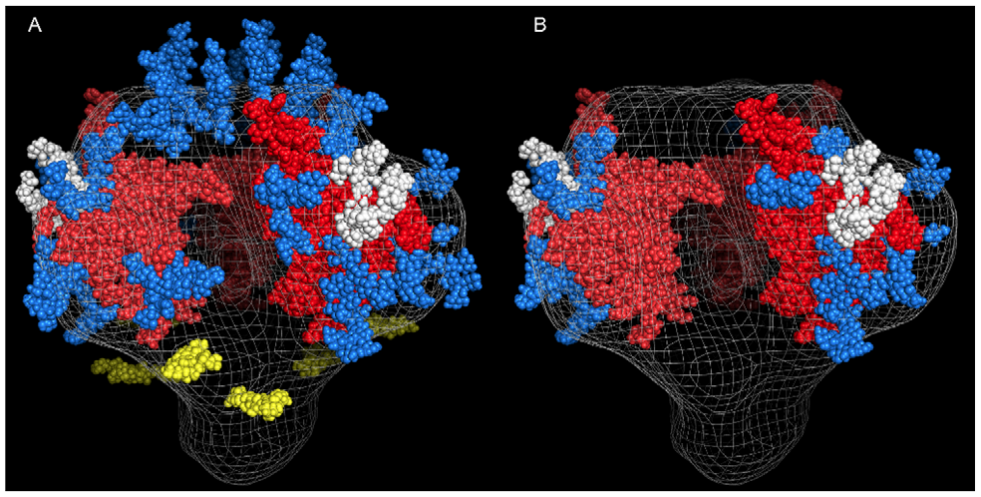
Model of glycosylation patterns of recombinant WT JRFL gp140 and partially deglycosylated gp140 under non-denaturing conditions. Shown are the images that were modeled based on the cryo-electron tomography as described by Schief [60]. Panel A: HIV-1 recombinant JRFL Env exhibits a number of high mannose glycans as determined by mass spectrometry (Figure 7 and Table S1). In this figure, the gp120 bearing the V3 loop [61] (PDB 2B4C)] trimer (red) is modeled over the cryo-EM structure (white mesh) (36), and its representative glycans are indicated in blue with those that are bound by the antibody 2G12 [37] in white. The fold of the prefusion gp41 trimer is not known, so gp41 glycans (yellow) have been positioned approximately equidistant around the stalk. Glycans bound to gp120 residues in the V1 and V2 loops have similarly been placed in reasonable locations across the top of the trimer. Depicted in Panel B are the glycans remaining after treatment with PNGase-F. In general, PNGase is able to remove the glycans that are attached to flexible parts of the gp120 structure, presumably allowing the enzyme to gain access to recognition elements and cleavage points. Glycans attached to Asn residues occuring in relatively stable structural elements of gp120 persist after PNGase-F treatment.

Based on the amino acid sequence of JRFL gp140 (including the signal peptide), all but four of the sites in the untreated protein were fully or partially occupied with glycans (Figure 7 and Table S1) (34). The unoccupied sites were: N141, N188, N611and N637. Upon extensive deglycosylation (500 U PNGase F per 1 µg of protein), the glycans at 12 out of 23 sites were removed: N88 (C1), N135 (V1/V2), N138 (V1/V2), N156 (V1/V2), N160 (V1/V2), N187 (V1/V2), N262 (C2), N276 (C2), N356 (C3) N462 (C4), N616, and N625 (Figure 7). The 10 sites on which glycosylation still remained were fractionally populated with glycans. Thus, PNGase F cleaved a fraction of the glycans at these sites, but the reaction was incomplete. Most of the conserved glycans were of the high mannose type and included those glycans involved in the 2G12 binding (N295, N332, N386) [33] (Figure 7). Figure 8 shows a model of JRFL gp140 Env before PNGase-F treatment (Figure 8A) and after PNGase-F treatment (Figure 8B) based on the cryo-EM structure (white mesh) of Liu *et al.* [36] and specific-site glycan analysis before PNGase-F treatment [34] and after treatment (Figure 7). Red molecules indicate gp120 amino acids, blue indicates gp120 glycans, white indicates 2G12 glycans [37], and yellow indicates gp41 glycans. Figure 8 depicts the loss of many complex glycans on gp120 and the loss of two gp41 glycans after PGNase-F treatment.

### Binding of mAbs 4E10 and 2F5 to gp140 Is to gp41 and Not to gp120

To determine if there were ancillary binding sites located on gp120, as previously suggested [38], binding of mAbs 4E10 and 2F5 to deglycosylated HIV-1 Env 89.6 gp120 was determined using the same non-denaturing conditions as used for binding of mAbs 2F5 or 4E10 mAb binding to deglycosylated JRFL gp140. We found that while sCD4 bound well to HIV-1 89.6 Env gp120 deglycosylated with up to 20 U PNGase F treatment, no binding of either mAbs 2F5 or 4E10 to gp120 was seen (Figure S2). Thus, the enhanced binding of mAbs 4E10 and 2F5 to the deglycosylated JRFL gp140 protein was indeed due to the binding to the gp41 component of gp140.

Next, we asked if absorption of 50 µg/ml of 4E10 by increasing amounts of either deglycosylated JRFL gp140 or deglycosylated HIV-1 Env 89.6 gp120 could absorb the 4E10 binding activity for the P-4E10 nominal peptide epitope.

As shown in Figure S3, addition of dilutions of absorbing JRFL Env gp140 protein to 50 µg/ml mAb 4E10 absorbed 4E10 binding activity to nominal 4E10 gp41 peptide, while addition of Env 89.6 gp120 (both WT glycosylated and PNGase F 20 U-treated) did not (Figure S3). Thus, the expressed epitope on JRFL gp140 is located on gp41 and PNGase F-treated gp140 absorbed 4E10 binding activity to the normal gp41 4E10 epitope peptide.

MAb 4E10 binding was the most enhanced of MPER antibodies to PNGase F-treated JRFL Env. To determine if the 4E10 neutralizing epitope was the target for 4E10 binding, we next asked if the nominal 4E10 binding epitope peptide (P-4E10: SLWNWFNITNWLWYIK) could absorb out the mAb 4E10 binding activity to deglycosylated JRFL gp140 (Figure S4). As shown in Figure S4, increasing amounts of the 4E10 epitope peptide absorbed the binding activity of 5 µg/ml mAbs 4E10 to the deglycosylated JRFL gp140.

### Analysis of Deglycosylated JRFL Env Binding to 2F5 and 4E10 Reverted Unmutated Ancestors (Putative B Cell Receptors of Naïve B Cells)

**Figure 9:**
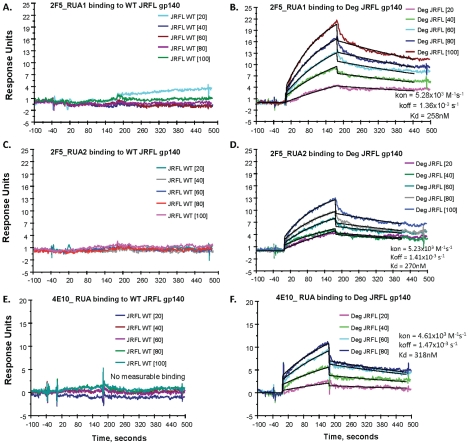
SPR measurements of binding of inferred RUAs of 2F5 and 4E10 to WT glycosylated and natively deglycosylated JRFL gp140 Env proteins. A dose range (from 20–100 µg/mL as indicated) of RUA1 (A–B) and RUA2 (C–D) inferred from the mutated 2F5 antibody sequence and one RUA (4E10 RUA, 20–80 µg/mL) (E–F) inferred from the 4E10 mutated antibody sequence were tested by SPR for binding to WT glycosylated (WT) and maximally deglycosylated (Deg) JRFL gp140 Env proteins immobilized on adjacent flow cells of the same sensor chip. Each antibody was captured on an anti-Ig Fc immobilized chip as described [7]. Neither of the RUAs of 2F5 (Panels 9A–9C) nor 4E10 (Panel 9E) bound to WT glycosylated JRFL gp140 Env protein, while reactivity of the RUAs of 2F5 (Panels 9B–9D) and 4E10 (Panel 9F) to deglycosylated JRFL gp140 Env proteins was present with the Kds and rate constants shown in each panel. Rate constants were derived by curve fitting analysis and using a 1∶1 Langmuir model.

While the somatically mutated 4E10 and 2F5 mAbs bound better to natively deglycosylated Env than to glycosylated Env, an immunogen needs to trigger the unmutated germline B cell receptors on the surface of naïve B cells [39], [40]. It has been previously demonstrated that a 2F5 RUA did not bind to fully glycosylated Env [16]. To assess Env binding to reverted unmutated antibody ancestors of the mutated mAbs 4E10 and 2F5, we used the program SoDA-2 [41] and Phylip's NAML [42] to infer RUAs of mAbs 2F5 and 4E10 resulting in two inferred RUAs of mAb 2F5 and one RUA of mAb 4E10 (Table S2 in SOM). These RUAs were produced in 293T cells as whole IgG1 antibodies by transfection and tested for binding to HIV-1 JFRL gp140 Env proteins. We found that indeed, the inferred RUAs of mAbs 4E10 and 2F5 did not bind to glycosylated JRFL gp140 Env, as previously shown for the 2F5 RUA by Xiao et al. [16]. However, both 2F5 and 4E10 RUAs did bind to natively deglycosylated JRFL gp140 Env oligomer (Figure 9). While the binding of the 2F5 RUAs to fully glycosylated JRFL gp140 Env was unmeasurable (Figures 9A and 19C), the binding Kds for partially deglycosylated JRFL gp140 Env were 258 nM and 270 nM, respectively (Figures 9B and 9D). Similarly, the binding of the 4E10 RUA for glycosylated Env was also unmeasurable (Figure 9E), while the binding Kd of the 4E10 RUA for the natively deglycosylated Env was 318 nM (Figure 9F). Thus, gp140 glycans mask or modulate the availability of MPER neutralizing epitopes for 2F5 and 4E10 RUA binding to gp41 linear neutralizing epitopes on the JRFL Env gp140 oligomers.

### Demonstration that Native Partial Deglycosylation of Another Env, the Group M Consensus Env, CON-S gp140, also Exposes the MPER Region and Enhances Binding of Deglycosylated Env to 2F5 and 4E10 RUAs

To determine if the effect of PNGase F on JRFL gp140 is generalizable to other Envs, we studied the effect of partial native deglycosylation on the group M consensus gp140 Env CON-S [29]. This Env has previously been reported to induce a degree of breadth among clade A, B and C Tier 1 virus strains [29]. We found that, like JRFL gp140 oligomers, PGNgase F 500 U treatment under native conditions of CONS gp140 resulted in reduced MW of monomers, dimers and trimers, and did not lead to Env degradation (Figure S1). Second, native deglycosylation of CON-S gp140 resulted in maintainance of sCD4 binding (Kd = 7.4 nM for CON-S glycosylated Env and Kd = 9.4 nM for deglycosylated Env) (Figure S5). Third, like deglycosylated JRFL gp140, deglycosylated CON-S gp140 Env could undergo sCD4 and A32 mAb-induced 17b binding to the CCR5 co-receptor gp120 binding site (Figure S6), and both mAbs 2F5 (Figures S7A and S7B) and 4E10 (Figures S7C and S7D) bound with lower Kds to deglycosylated CON-S gp140 when compared to WT glycosylated CON-S gp140.

Interestingly, the WT glycosylated CON-S gp140 constitutively bound (before deglycosylation) to both 2F5 RUAs 1 and 2 (Figures S8A and S8C), demonstrating that some glycosylated recombinant Envs do have the ability to bind to broadly neutralizing antibody germline antibodies. 2F5 RUA1 bound better to glycosylated CON-S gp140 than did 2F5 RUA2 (Kd = 118 nM for RUA1 vs Kd = 4.0 µM for RUA2). The enhancement of binding to the deglycosylated CON-S was only two-fold for RUA1 (Kd = 67 nM) while the enhancement of binding of 2F5 RUA2 for deglycosylated CON-S gp140 Env was an order of magnitude (Kd = 4.0 µM for WT glycosylated CON-S gp140 and Kd = 0.36 µM for deglycosylated CON-S Env).

For CON-S gp140, the pattern of binding to the 4E10 RUA was similar to that of JRFL gp140, with no measurable binding of glycosylated CON-S gp140 to the 4E10 RUA, while the deglycosylated CON-S gp140 bound to the 4E10 RUA with a Kd of 0.23 µM (Figures S8E and S8F). That WT glycosylated CON-S gp140 binds to the RUAs of 2F5 in this setting is not surprising since we have previously published that CON-S gp140 produced in 293T cells (as the CON-S gp140 was produced in this study) is not glycosylated in gp41 [34]. That the expression of the epitopes for both mAbs 2F5 and 4E10 are further enhanced on deglycosylated CON-S therefore suggests that removing glycans on gp120 can also affect exposure of MPER neutralizing epitopes. Regardless, the effect of PNGase F native deglycosylation is not unique to JRFL gp140 Env.

### Immunogenicity of HIV-1 JRFL gp140 and Deglycosylated JRFL gp140 Env Proteins in Rhesus Macaques

**Figure 10:**
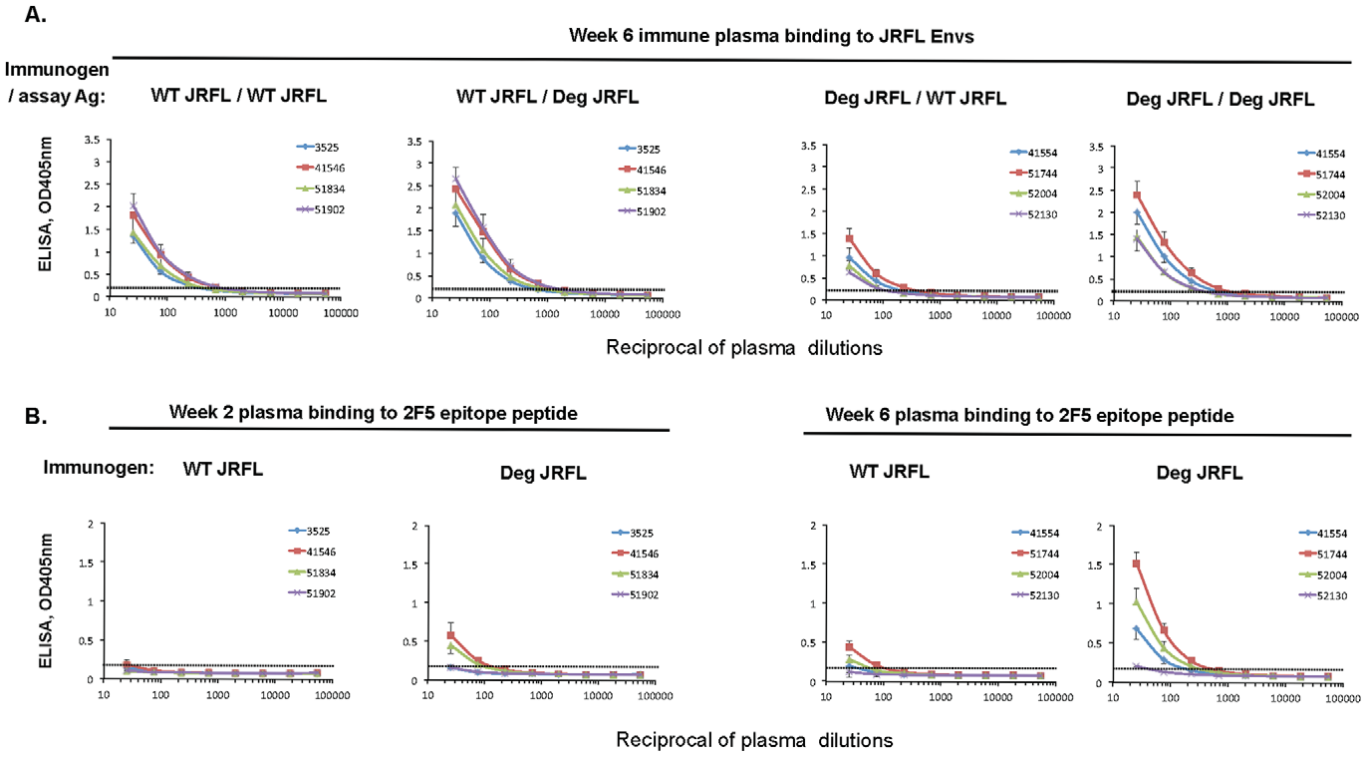
Enhanced immunogenicity of deglycosylated JRFL gp140 Env as determined by plasma levels of 2F5 epitope binding antibodies in immunized rhesus macaques. Rhesus macaques (4 per group) were immunized at weeks 0 (prime) and 4 (boost) with 100 ug of either glycosylated (WT) or natively deglycosylated (Deg) JRFL gp140 Env. Plasma samples were collected at weeks 2 and 6 from each group (as indicated on the top of the panels) and were assessed for binding to WT JRFL gp140 Env and Deg JRFL gp140 Env (Panel 10A). The antibody responses to wild-type JRFL Env or to Deg JRFL Env were undetectable after the prime and are not shown. The responses to WT JRFL Env and Deg JRFL Env after the week 4 boost (the week 6 blood draws) are shown. The antibody response to 2F5 nominal MPER peptide (QQEKNEQELLELDKWASLWN) after the prime (week 2) and boost (week 6) is shown (Panel 10B). Shown on the x axis are the reciprocal dilutions of plasma of the immunized moneys used in ELISA. The dotted lines indicate the negative cut-off values. The level of 2F5 epitope antibody response at the initial dilution (1∶25) in week 6 plasma were higher in animals immunized with deglycosylated JRFL Env than in animals immunized with WT JRFL Env (p = 0.035, Student's t test). Data shown are mean values +/− standard deviation derived from 3 separate experiments performed.

To determine if the enhanced binding of mAbs 4E10 and 2F5 and their RUAs to deglycosylated HIV-1 JRFL gp140 Env might have resulted in enhanced immunogenicity for induction of MPER antibodies, 4 rhesus macaques per group were immunized with either deglycosylated JRFL Env gp140 or WT JRFL gp140. Plasma samples were obtained 2 weeks after the first priming and second boosting immunizations and evaluated for binding antibody to the immunizing Env and to 2F5 and 4E10 nominal membrane proximal external region (MPER) peptides. Both the deglycosylated HIV-1 JRFL gp140 Env protein and WT JRFL gp140 Env were immunogenic and induced similar levels of antibodies to the immunizing Envs after 2 immunizations (Figure 10A). Whereas the WT glycosylated JRFL gp140 Env induced minimal levels of antibodies after the first and second immunizations to a 2F5 epitope peptide (QQEKNEQELLELDKWASLWN), in contrast, the deglycosylated JRFL gp140 Env induced enhanced levels of 2F5 epitope antibodies after the second immunization in 3 of 4 animals (Figure 10B) (p = 0.035, Student's t test). However, neither the deglycosylated HIV-1 JRFL Env proteins nor WT JRFL gp140 Env induced antibodies to HIV-1 MPER 4E10 epitope peptide, SLWNWFNITNWLWYIK (data not shown). Finally, while antibodies were present in both groups that neutralized the SF162.B pseudovirus, neither group neutralized the 2F5-sensitive HIV-1 Env BG1168.B pseudovirus (data not shown), indicating that the induced rhesus MPER antibodies either were non-neutralizing or were of insufficient titers to neutralize. Nonetheless, these data demonstrate proof-of-concept of enhanced 2F5 epitope immunogenicity of native deglycosylated JRFL gp140 Env.

## Discussion

We have demonstrated that non-denaturing (native) deglycosylation of HIV-1 JRFL and the group M consensus gp140 envelope oligomers with PNGase F resulted in enhanced Env binding to gp41 broadly neutralizing mAbs 4E10 and 2F5, and as well, induced or enhanced binding of gp140s to 2F5 and 4E10 reverted unmutated ancestor antibodies. Moreover, immunization of the JRFL deglycosylated gp140 Env resulted in enhanced antibody induction to the gp41 2F5 peptide epitope.

Treatment of Env with glycosylation inhibitors and neuraminidase has been reported to enhance HIV-1 binding and neutralization by mannose-binding lectin, and to facilitate immunocapture of virions by both mAbs 2G12 and 2F5 [43]. Thus, the selective removal of complex glycans by non-denaturing PNGase F treatment may similarly expose the 2F5 and 4E10 MPER binding site for enhanced mAbs 2F5 and 4E10 binding. MAb 2G12 has been reported to bind to gp120 N-linked high mannose residues at N295, N332, and N392, as well as with peripheral glycans from 386 and 448 [3], [37]. Interestingly, our mass spectrometry analysis of deglycosylated JRFL gp140 demonstrated selective loss of N-linked glycans particularly around V1 and V2 with high mannose residues at N295, N332, and N392 remaining intact [34], [35]. The non-denatured deglycosylated JRFL gp140 Env bound well to mAb 2G12 and also bound to DC-SIGN. In this regard, the gp120 high mannose residues involved in 2G12-gp120 binding have been reported to be involved in gp120-DC-SIGN binding [44]. Thus, selective removal of gp140 glycans may induce conformational changes or unmasking of the gp41 MPER 4E10 epitope that results in enhanced on-rate (ka) of mAbs 4E10 and 2F5 binding.

Walker et al. have recently reported that 25% chronically infected HIV-1 subjects with broad neutralizing antibodies had neutralizing plasma antibody activity targeted at Env glycans at N332, and at N295 in some subjects [45]. Although N332 and N295 glycans are involved in mAb 2G12 binding, their N332 and/or N295 activity was found to bind a glycan epitope distinct from that of 2G12 [45]. That PNGase treatment of HIV-1 Env under non-denaturing condition retained all N-linked glycans involved in 2G12 and in the novel N295/N332 neutralizing epitopes, suggests natively deglycosylated HIV-1 gp120 or gp140 Env may be of use in vaccine formulations targeted at elicitation of anti-glycan gp120 neutralizing antibodies.

Yuste *et al.*, has demonstrated that mutations in gp41 N-linked glycosylation sites exposed a neutralization epitope N-terminal to the MPER [23]. From Yuste *et al.*, we would predict that cleavage of N-linked glycans at 611 616, 625 and/or 637 might also be responsible for directly unmasking the 2F5 and 4E10 gp41 epitopes, or inducing conformational changes of the Env that indirectly expose the gp41 membrane proximal external region, or both. Indeed, three out of four glycosylation sites in gp41 were occupied in recombinant untreated JRFL gp140, and were removed in natively deglycosylated JRFL gp140 (Figure 7). Additionally, we and others have previously shown that enhanced exposure of the MPER epitope by single amino acid changes can increase the binding and neutralizing activity by broadly neutralizing MPER antibodies [46]–[48].

Since the 4E10 antibody is markedly polyreactive [6], it was important to consider that the 4E10 epitope exposed on PNGase F-treated Env gp140 might be distant from the ^672^WTDIT-NWLWY^681^ gp41 4E10 nominal epitope. Hages-Braun *et al*. have suggested that two ancillary 4E10 binding sites are located on gp120 at ^34^ LWVTVYYGVPVWK^46^ and on gp41 at ^512^AVGIGAVFLGFLGAAGSTMGAASMTLTVQAR^542^ [38]. However, our studies demonstrated no 4E10 binding to PNGase F-treated gp120, and PNGase F-treated gp140, but not gp120, absorbed the binding of mAb 4E10 to the gp41 peptide (P-4E10, SLWNWFNITNWLWYIK). These data suggested that the reason that mAbs 2F5 and 4E10 RUAs can bind PNGase-treated JRFL gp140 is most likely due to enhanced exposure via glycan unmasking of the gp41 MPER, or an induced gp41 MPER conformational change that allowed for a faster on-rate for mAbs 2F5 and 4E10 to bind to gp41. That most of the deglycosylated forms of JRFL and CON-S gp140 are oligomeric and only a minor species is monomeric for both Envs (Figure S1), demonstrates that the exposure of the MPER is on oligomeric Env forms.

A number of studies have suggested that deglycosylation can affect HIV-1 envelope antigenicity and immunogenicity [17], [19], [21]–[23], [49]–[54]. A non-glycosylated outer domain of gp120 has been reported to be immunogenic and induce weak neutralizing antibodies in rabbits [50]. For example, loss of sialic acids on gp120 has been reported to improve Env immunogenicity [49], [55]. Strategies for native deglycosylation of Env have been described that can improve both immunogenicity and sensitivity of resulting virions to neutralization [51], [53].

Frey *et al.* have reported weak or no binding of recombinant HIV-1 Envs to mAbs 2F5 and 4E10, but strong binding of these antibodies to a stabilized gp41 intermediate molecule [15]. Whether the epitope on natively deglycosylated recombinant JRFL gp140 is in the intermediate conformation of gp41 seen by mAbs 2F5 and 4E10 during the infection process is not known. However, we have recently shown that glycosylated JRFL gp140 Env can prime for an MPER peptide-liposome boost and induce antibodies focused on the DKW core 2F5 epitope that bind to the gp41 intermediate epitope (Sekaran, M. Alam, SM, Haynes, BF, unpublished). That deglycosylated JRFL gp140 is more immunogenic in rhesus macaques than glycosylated JRFL for 2F5 epitope MPER antibodies suggests that deglycosylated JRFL may have enhanced immunogenicity as a prime in this prime-boost setting.

Other factors in addition to the conformation of the MPER neutralizing epitopes appear to contribute to regulation of anti-MPER broad neutralizing antibodies. For example, we now have experimental data with mAbs 2F5 and 4E10 knock-in mice that B cells expressing these VHs are controlled by both central and peripheral tolerance mechanisms in these animals [13], [14]. Thus, immunogen design for optimal neutralizing epitope exposure is only one-component of the problem for inducing anti-MPER antibodies. Nonetheless, the increased affinity of the non-denaturing PNGase F-treated gp140 Env may render it a preferred Env immunogen in the setting of formulation of Envs with potent adjuvants that are designed to circumvent peripheral tolerance [56], [57].

While the RUAs in this paper are close approximations of the unmutated ancestor B cell receptors on naïve B cells, it should be noted that they are only inferred and may not precisely mimic the binding of membrane bound IgM B cell receptors on naïve B cells. Our data demonstrate that RUAs of both mAbs 2F5 and 4E10 can recognize the MPER well in the context of natively deglycosylated JRFL gp140, but not in fully glycosylated JRFL gp140. Interestingly, the glycosylated group M consensus Env CON-S gp140 constitutively bound to both 2F5 RUAs. This finding is consistent with our previously published site-specific glycan analysis of the CON-S gp140 in which we found, unlike JRFL gp140, that CON-S gp140 when expressed in 293T cells, has no occupied gp41 glycan sites [34]. That native deglycosylation of CON-S gp140 still resulted in enhanced binding to unmutated ancestors of 2F5 and 4E10 suggests that removal of gp120 glycans modulates access to gp41 as well. Thus, the inability of mAbs 2F5 and 4E10 reverted unmutated ancestors to react with recombinant HIV-1 gp140 Envs in some instances may be due to glycan masking or glycan modulation of conformations of MPER neutralizing epitopes, or may be due to “holes” in the native B cell repertoire for glycosylated HIV-1 Env. That the MPER 2F5 and 4E10 gp41 epitopes can be exposed with native deglycosylation, to us suggests the importance of glycan masking on glycosylated gp140 to 2F5 and 4E10 unmutated ancestor antibody non-reactivity. In light of the host immunoregulatory controls that also appear to play roles in control of MPER broad neutralizing antibody induction, both structural considerations of immunogen design targeted to optimized binding to reverted unmutated ancestor antibodies, in concert with methods of immunogen formulation to access and drive MPER-specific naïve and memory B cell proliferation likely will be required for the ultimate safe induction of MPER broad neutralizing antibodies.

## Materials and Methods

### Cloning and Expression of HIV-1 Env Proteins

A codon-optimized gene encoding the gp140CF (C  =  gp120-gp41 cleavage site deleted, F  =  fusion domain-deleted) of HIV-1 WT JRF Env in plasmid pR-JRFL140 (kindly provided by Feng Gao, M.D., Duke, Durham, North Carolina) was subcloned to pcDNA3.1(-)/Hygro eukaryotic expression vector (Invitrogen, Carlsbad, CA) via *Xba* I site at the 5′ end and a *BamH*I site at the 3′ end under the regulation of the CMV immediate early promoter. The resulting recombinant plasmid, pcDNA/H-JRFL 140, was linearized by restriction endonuclease *Ssp* I (Invitrogen, Carlsbad, CA) digestion. Two µg of purified plasmid was transfected into HEK293T cells (ATCC, Bethesda, MD) at approximately 80% confluence with LipoFectamine 2000 (Invitrogen, Carlsbad, CA) per the manufacturer's protocol. Two days post transfection, cells were digested with trypsin-EDTA, resuspended with DMEM HG media (Sigma, St. Louis, MO) containing 20% fetal bovine serum (DMEM-20) and 200 µg/mL of hygromycin B (Sigma) (selection media), and dispersed to 10–20 100 mm polystyrene Petri dishes (Becton-Dickinson, Franklin Lakes, NJ). Hygromycin B-resistant colonies were picked, digested and re-suspended in selection media. Stably transfected cells were cloned by limiting dilution and adapted in serum-free media Pro293A (BioWhittaker, Switzerland), and used for production of purified recombinant WT JRFL gp140 protein using lectin agarose beads (*Galanthus Nivalis*, Vector Laboratories, Burlingame, CA) and stored at −80°C until use [29]. The group M consensus gp140 Env, CON-S gp140 CFI (C  =  gp120-gp41 cleavage site deleted, F  =  fusion domain-deleted, I  =  immunodominant gp41 region deleted) protein was expressed and produced as previously described [29].

### Cells and Viruses

DC-SIGN-expressing NIH 3T3 cells, parent NIH 3T3 cells, TZM-BL cells that express CD4/CCR5/CXCR4 were obtained from NAID/NIH AIDS Reagent Repository. Cell lines were grown in Dulbecco's modified Eagle's medium (DMEM, Life Technologies/Invitrogen) supplemented with 10% heat-inactivated fetal bovine serum (FBS).

### Monoclonal Antibodies (mAbs) and Soluble CD4 (sCD4) Human

MAbs known to bind epitopes on gp120 (A32) [31] the gp120 V3 loop (F39F), and the CCR5 binding site (17b) [30], were kindly provided by James Robinson (Tulane Medical School, New Orleans, LA). Anti-gp41 membrane proximal external region (MPER) mAbs 2F5, and 4E10, and 2G12 [33] were purchased from Polymun Scientific, Vienna, Austria. T8 is a murine mAb that binds to the gp120 C1 region (a gift from P. Earl, NIH). Mouse mAb 3B3 binds near the N-terminus of gp120 and has broadly cross reactivity with HIV-1 gp120 proteins of different subtypes [32]. Mouse anti-DC-SIGN monoclonal antibodies [58] and recombinant sCD4 were obtained from the NIAID.NIH AIDS Reagent Repository. MAb 1b12 against the CD4BS was the gift of Dennis Burton, Scripps, La Jolla, CA [20].

### Deglycosylation of Proteins

Peptide-N-glycosidase F (PNGase F) that removes N-linked carbohydrates was purchased from New England Biolabs (Beverly, MA). Progressive native deglycosylation was performed using 100 µg of HIV-1 JRFL Env protein with varied amounts of PNGase F ranging from 5, 10, 20, 50, 100 and 500 units (U) per µg of HIV-1 JRFL gp140 Env protein. For each experiment, 100 µg of Env protein were digested with indicated amount of PNGase F in 200 µL of volume (resulting in the protein concentration of 0.5 µg/µL). The reaction was carried out at 37°C for 24 h and then stopped by freezing the reaction mixtures at −80°C. Deglycosylation in denaturing conditions was performed by digesting every 100 µg of pre-denatured protein in denaturing buffer (0.5% SDS and 1.0% β-ME) at 100°C for 10 min with 1 µL (500 U) of PNGase F at 37°C for 2 h using the protocol as recommended by the manufacturer. Deglycosylation of the JRFL Env protein under the native condition was optimized by either prolonging the incubation time or increasing the amount of enzyme used in order to achieve the maximal deglycosylation. In the former case, the incubation time extended up to 7 days only minimally improved the extent of native deglycosylation of gp140 (data not shown). On the other hand, the prolonged incubation increased the risks for protein to be degraded. In contrast, with the fixed reaction time (approximately 24 h), the increased amounts of the enzyme dramatically increased the completeness of the native deglycosylation of Env. Deglycosylation under non-denaturing conditions with PNGase F (500 U per µg of JRFL Env) reduced the size of the deglycosylated JRFL gp140 Env protein close to the theoretical polypeptide size (approximately 80 kDa) as analyzed by SDS-polyacrylamide gel electrophoresis (SDS-PAGE), which indicated that approximately 80% of carbohydrates have been removed. Deglycosylation of CON-S gp140CFI was performed under non-denaturing conditions using PNGase F at 500 U per µg of Env protein.

### SDS-PAGE and Western Blot Analysis of the Env Proteins

Untreated and deglycosylated HIV-1 JRFL gp140CF and CON-S gp140CFI Env proteins were fractionated on 4–12% Bis-Tris SDA-PAGE gels (Invitrogen, Carlsbad, CA) under reducing (4% Beta-mercaptoethanol, β-ME, Fisher Scientific, Fair Lawn, NJ) or non-reducing conditions, stained with coomassie blue dye, or transferred onto nitrocellulose filters and probed with anti-Env human mAbs 17b, 1b12, 2F5, 2G12, 4E10, A32, F39F. Goat anti-human IgG alkaline phosphatase (AP) conjugates (Sigma Aldrich, Inc, St. Louis, MO; 1∶3,000 dilution) was used as secondary antibodies in Western-blots.

### Surface Plasmon Resonance (SPR)

SPR assays using a BIAcore 3000 instrument were carried out to characterize the binding of untreated and deglycosylated HIV-1 Env proteins to anti-HIV-1 MPER mAbs 2F5 and 4E10 as well as to their inferred reverted unmutated ancestors. Data analysis was performed with BIAevaluation 3.0 software (BIAcore Inc, Upsaala, Sweden). To measure the affinity (Kd) and kinetics of the interaction of the Env proteins with mAbs, the Env glycoproteins at varying concentrations were flowed over mAb 2F5 or 4E10 that were captured on anti-Fc IgG immobilized on the sensor chip CM5 with amine coupling kit (Biacore AB) and as described earlier (7, 8, 59). The binding data were recorded in real time at 25°C with a continuous flow of phosphate-buffered saline (150 mM NaCl, 0.005% surfactant P20 [pH 7.4]) at 30 µL/min following subtraction of non-specific signal over the control mAb surface [7], [8], [59].

### ELISA

EIA plates (Costar 3590, Corning, NY) were coated with JRFL gp140 protein (0.2 µg/well) in 100 µL of carbonate-bicarbonate (CBC) buffer (pH 9.6) over night at 4°C. Plates were then blocked with CBC containing 3% BSA (Sigma, St. Louis, MO) for 2 h at room temperature (RT). To evaluate the antibody binding kinetics, serial dilutions of each mAb (17B, 1b12, 2F5, 2G12, 4E10, and F39F) starting from 10 µg/ml in 50 µL in dilution buffer (PBS, 0.05% Tween-20, 2% goat serum and 3% BSA) were added to the plate followed by the incubation at RT for 1 h. After washing with dilution buffer, goat anti-human IgG-AP (Sigma, St. Louis, MO) was added and incubated in the same way to detect the binding of human antibodies to Env proteins. Alternatively, equal volume of native deglycosylation and mock deglycosylation reaction solutions containing equal amount of initial Env protein were serially diluted in CBC buffer (pH 9.6) starting from 10 µg/mL (2-fold serial dilution for 12 point titrations). One hundred (100) µl of the diluted solution were coated on the 96-well EIA plate (Costar 3590, Corning, NY) each well and incubated at 4°C overnight and then blocked with CBC containing 3% BSA (Sigma, St. Louis, MO) for 2 h at RT.

Each human mAb was diluted in buffer (PBS, 0.05% Tween-20, 2% goat serum and 3% BSA) to 1.0 µg/mL and 100 µl per well of each diluted antibody was incubated at RT for 1 h. For sCD4, 100 µl of 0.2 µg/mL was used. Goat anti-human IgG-AP (Sigma) was used to detect the binding of human antibodies to Env proteins. For detection of binding of sCD4, biotin-labeled anti-CD4 mAb (0.1 µg/mL in 100 µl/well) and the Strepavidin (Promega, 1∶1000, 100 µl/well) were used. In selected experiments, Env oligomers were captured on plates pre-coated with mAb 3B3 and then tested for mAb reactivity as above. ELISA results were read at 405 nm using ELISA radar (Bio-Rad, Berkeley, California).

Rhesus macaque binding antibodies to HIV-1 WT glycosylated JRFL gp140 Env and deglycosylated JRFL gp140 Env were assayed by indirect ELISA. HIV-1 WT glycosylated JRFL gp140 Env and deglycosylated (Deg) JRFL gp140 Env, 2F5 nominal MPER epitope peptide SF62 (QQEKNEQELLELDKWASLWN) and 4E10 eiptope peptide P-4E10 (SLWNWFNITNWLWYIK) were coated at 200 ng/well in 96-well ELISA plates (4 degrees C) followed by incubation with dilutions of immune plasma. AP conjugated goat anti-monkey IgG heavy- and light chain specific antibody was used as seconday antibody.

### DC-SIGN Binding Assay

To assess the reactivity of untreated and deglycosylated proteins to bind DC-SIGN, WT glycosylated and deglycosylated JRFL gp140 proteins were chemically labeled with the Alexa Fluor 647 dye (AF647) using a protocol as recommended by the manufacturer (Invitrogen, Carlsbad, CA). NIH 3T3/DC-SIGN or the parent NIH 3T3 cells (5×10^5^ cells in 100 µL of PBS containing 1% BSA in the presence of 1 mM Ca^++^ and Mg^++^ ions) were incubated with 1.0 µg/mL of the AF647-labeled undeglycosylated or deglycosylated JRFL gp140 proteins at 4°C for 30 min. The cells were washed for 4 times with the PBS buffer. The cells were resuspended in 400 100 µL of PBS buffer and analyzed with an LSRII flow cytometer (Becton Dickinson) [58]. CD4 expressing TZM-b1 cells were used as a positive control for binding of the AF647-labeled undeglycosylated and deglycosylated JRFL gp140 proteins.

### Analysis of Carbohydrates by Mass Spectrometry

We have previously analyzed and compared JRFL gp140CF and CON-S gp140CFI Envs for glycan site-occupation and content [34]. To determine the glycosylation profile of both native and the deglycosylated JRFL gp140 Env proteins, the latter were treated with proteases and the digestion products were analyzed by LC/MS, as described previously and below [34], [35].

Deglycosylated JRFL gp140 Env envelope proteins were either treated with Endo H or (separately) Endo F3, to reduce the glycan heterogeneity to a single N-acetlyhexosamine, attached to the glycosylation site. Core fucosylated residues also remained fucosylated in this procedure. Typical conditions included incubating the Env (approximately 7 fmol) with enzyme, at a ratio of at least 30 U/mg of Env protein. For Endo H deglycosylation experiment, the sample was denatured with 2 M urea in 100 mM tris buffer (pH 5.5) followed by the addition of 2 µL of Endo H. After thorough mixing, the reaction was incubated for 48 hours at 37°C. For Endo F3 deglycosylation experiment, samples were incubated with 6 µL of 250 µM NH_4_C_2_H_2_O_2_ (pH 4.5) and 2 µL of Endo F3 for two weeks at 37°C.

After deglycosylation, proteins were split into two fractions and subjected to enzymatic digestion using trypsin and Glu-C. Prior to enzyme addition, proteins were unfolded using 6 M urea, and reduced and alkylated at room temperature using 10 mM DTT and 15 mM IAA at RT, respectively. Digestion conditions were carried out in tris buffer with 3 mM EDTA overnight at 37°C. For tryptic digest, pH was 8.5 and protein to enzyme ratio was 30∶1; for Glu-C digest, pH was 7.8 and the protein to enzyme ratio was 20∶1. Both reactions were quenched with the addition of 1 µL glacial acetic acid. The digestion protocols were performed three times on different days, with the same initial batch of protein, to ensure reproducibility and reliability. As an additional control experiment, WT glycosylated protein was subjected to the same protocols to verify that the method would effectively detect all sites on the protein that remained glycosylated.

For carbohydrate analysis, peptides were detected by LC-MS, using a Dionex UltiMate capillary LC system (Sunnyvale, CA) equipped with a FAMOS well plate autosampler; adjoined to a Fourier transform ion cyclotron resonance mass spectrometer (LTQ-FT, ThermoScientific, San Jose, CA). Separation of the sample (5 µL) was achieved using a C18 PepMap 300 column (300 µm i.d. ×15 cm, 300 Å, LC Packings, Sunnyvale, CA) at a flow rate of 5 µL/minute. Separation was achieved using a linear gradient over 65 minutes, and the eluted peptides were ionized using a 2.8 kV source voltage for ESI. High resolution MS and low resolution MS/MS data were collected using a data-dependent scanning protocol, where the five most intense ions were selected for MS/MS, after each high resolution MS run, and those ions were subsequently dynamically added to an exclusion list for 3 minutes. Peptides (both deglycosylated, and those with remaining glycosylation) were detected using the MS and MS/MS data with the assistance of a Mascot search (Matrix Science, London, UK, version 2.2.04), after inputting a custom HIV Env database. All automated assignments were further verified by manually assigning the relevant MS/MS spectra. All glycosylation sites were detected as glycosylated or deglycosylated, except for N406, which was not detected by MS analysis.

### Ethics Statement

This study involved in the use of rhesus macaques was carried out in strict accordance with the recommendations in the Guide for the Care and Use of Laboratory Animals of the National Institutes of Health in BIOQUAL (Rockville, MD.) BIOQUAL is fully accredited by AAALAC and through OLAW, assurance number A-3086. The protocol was approved by the BIOQUAL IACUC. All physical procedures associated with this work were done under anesthesia to minimize pain and distress. Four rhesus macaques per group were immunized intramuscularly with 100 ug/dose of HIV-1 JRFLgp140 Env wild-type (WT) or JRFL gp140 CF Env deglycosylated using PNGase F at 500 U/µg Env using AS01B adjuvant (generously provided by Gerald Voss, GlaxoSmithKline Biological, Rixensart, Belgium) at week 0 and 4. Blood samples were collected at week 2 and week 6 for isolation of immune plasma.

## Supporting Information

Figure S1 **Analysis of deglycosylated JRFL protein in blue native (BN) gel and SDS-PAGE.** JRFL and CON-S gp140 Env and JRFL gp140 and the CON-S gp140 Env proteins that were natively deglycosylated using 500U PNGase F/ug Env were fractionated in either blue native gel (Panel S1A) or in 4-12% SDS-PAGE under reducing condition and stained with coommassie blue (Panel S1B). The + and – above the individual lanes indicate that JRFL gp140 or CON-S gp140 were treated (+) or not treated (-) with 500U PNGase F/µg Env. In BN-PAGE gel analysis, deglycosylated JRFL gp140 and CON-S gp140 Envs migrated as monomer, dimer and trimer with decreased molecular weight as results from the removal of glycans compared with the WT glycosylated Envs (panel S1A). Panel B shows that in SDS-PAGE under reducing conditions, treatment with 500U/ug Env PNGase F reduced the molecular weight to approximately 80 kDa for JRFL gp140 CF Env and 75kDa for CON-S gp140 CFI Env. The slightly lower molecular weights of protein bands of CON-S gp140 were due to the deletion of the immunodominant region in the CON-S gp140CFI design compared with JRFL gp140CF protein without deletion of the immunodominant region [29]. Note that in the reduced Coomasie stained SDS-PAGE gels (Panel S1B) there are no bands higher than gp140, indicating compete reduction of the Env dimers and higher MW forms.
(TIF)

Figure S2 **Binding of mAbs 2F5 and 4E10 to deglycosylated Env gp120 proteins.** Shown is the evidence of lack of binding in ELISA of mAbs 2F5 and 4E10 to Env gp120 proteins directly coated on ELISA plates after progressive deglycosylation from 0 to 500 U per µg of Env protein (DEG 0-DEG 500). Binding of sCD4 to Env gp120 in this assay setting was maintained on Env gp120 protein up to 100 U of PNGase F.
(TIF)

Figure S3 **Inhibition of binding of mAb 4E10 to 4E10 binding epitope peptide (P4E10) by WT glycosylated and deglycosylated JRFL 140 protein.** MAb 4E10 at 50 µg/ml was first pre-incubated with the indicated concentrations (in x-axis) of HIV-1 Env protein of either WT glycosylated (Deg 0) or moderately deglycosylated (Deg 20, 20 U per µg of protein) JRFL gp140CF or Env gp120, and then assayed for binding to a 4E10 epitope peptide (SLWNWFNITNWLWYIK) by ELISA. Incubation of either WT (Deg 0) or moderately deglycosylated (Deg 20) Env gp120 had little effect on the binding of mAb 4E10 to the 4E10 epitope peptide, while deglycosylated JRFL gp140 protein showed more efficient absorption than did WT glycosylated JRFL Env protein of the binding of mAb 4E10 to the 4E10 epitope peptide.
(TIF)

Figure S4 **Inhibition of mAb 4E10 binding to deglycosylated JRFL gp140 proteins.** MAb 4E10 was pre-incubated with indicated concentrations (x-axis) of 4E10 epitope peptide (SLWNWFNITNWLWYIK) and then assayed in ELISA for binding to deglycosylated (500 U PNGase F) JRFL gp140 Env protein. Percentage of binding (y axis) of absorbed mAb 4E10 to the Env antigen was determined in comparison with the binding of non-absorbed antibody and plotted versus the amount of 4E10 peptide used for absorption. 4E10 peptide (SLWNWFNITNWLWYIK) inhibited the binding of 4E10 mAb to deglycosylated JRFL gp140 in a dose-dependent manner. Data shown are representative of three experiments performed.
(TIF)

Figure S5 **Binding of soluble (s) CD4 to WT glycosylated and deglycosylated CON-S gp140 by SPR.** Surface plasmon resonance assays were performed as described in Materials and Methods. Shown are the binding of sCD4 to the WT glycosylated (WT) (Panel S5A) and deglycosylated (Deg) (Panel S5B). sCD4 was covalently immobilized to a CM5 sensor chip (BIAcore), and WT glycosylated and deglycosylated CON-S gp140 injected over each surface (5-20ug/mL and 40-100 µg/mL, respectively). Rate constants and Kd measurements were made using the 1∶1 Langmuir model. The on-rate (K_on_), off -rate (K_off_) and Kd are indicated in the individual panels. Each analysis was performed at least twice.
(TIF)

Figure S6 **Analysis of antigenic epitopes expressed on WT glycosylated and deglycosylated CON-S gp140 by surface plasmon resonance (SPR).** SPR assays were performed as described in Materials and Methods. Shown is the ability of WT glycosylated and deglycosylated CON-S gp140 to bind to mAb 17B (Panel S6B). sCD4 or HIV-1 mAbs T8 and A32 were covalently immobilized to a CM5 sensor chip (BIAcore), and WT glycosylated and deglycosylated CON-S gp140 were injected over each surface to capture 320 – 500 RU of Env proteins. To determine induction of 17b MAb binding to glycosylated and deglycosylated CON-S gp140, Env proteins at equivalent RU amounts were captured on individual flow cells immobilized with sCD4 or mAb A32 or T8. Following stabilization of each of the surfaces, varying concentrations of mAb 17b (25 to 100ug/ml) was injected and allowed to flow over each of the immobilized flow cells as illustrated in the diagram above the SPR profiles in Panel B. The on-rate (k_on_), off -rate (k_off_) and Kd are indicated in individual panels. Each analysis was performed at least twice.
(TIF)

Figure S7 **SPR assays of binding of mAbs 4E10 and 2F5 to WT glycosylated and deglycosylated CON-S gp140 Envs.** MAbs 2F5 (Panels S7A, S7B) and 4E10 (Panel S7C, S7D) were bound to anti-human Ig Fc immobilized on the CM5 sensor chip. Then varying concentrations (ranging from 20 to 100 µg/ml) of glycosylated (WT) or deglycosylated (Deg) Con S gp140 (20-100 µg/ml or 1 to 40 ug/mL as indicated) proteins were injected over the mAbs on the chip and binding kinetics were recorded. Rate constants and Kd measurements were made using the 1∶1 Langmuir model and data are as indicated. Deglycosylation of CON-S gp140 enhanced 2F5 and 4E10 Kds by ∼4 fold and ∼7-fold, respectively. Each analysis was performed at least twice.
(TIF)

Figure S8 **SPR measurements of binding of inferred RUAs of 2F5 (S8A-S8D) and 4E10 (S8E and S8F) to WT glycosylated and deglycosylated CON-S gp140 Envs.** Dose ranges (20-100 µg as indicated) of RUA1 (Panels S8A and S8B) and RUA2 (Panels S8C and S8D) inferred from mAb 2F5, and one RUA (Panels S8E and S8F) inferred from mAb 4E10 were tested by SPR for binding to glycosylated (WT) and deglycosylated (Deg). Each antibody was captured on an anti-Ig Fc immobilized chip as described earlier [7], [8], [59]. Rate constants and Kd measurements were made using the 1∶1 Langmuir model. The on-rate (K_on_), off -rate (K_off_) and Kd are indicated in individual panels. Each analysis was performed at least twice.
(TIF)

Table S1 **Mass spectrometry analysis of deglycosylated JRFL gp140 Env treated with Endo H and Endo F3.**
(DOC)

Table S2 **Variable region sequence of mAb 2F5 and 4E10 as well as their inferred reverted and unmutated heavy- and light-chain genes.** Shown is the summary of MS data. Each peptide containing a potential glycosylation site was detected as either a glycopeptide, a deglycosylated peptide, or as a nativly nonglycosylated speices. Original regions of HIV-1 Env for the detected individual peptides are indicated.
(DOC)
