# Supplementary figures and images for: Envelope Deglycosylation Enhances Antigenicity of HIV-1 gp41 Epitopes for Both Broad Neutralizing Antibodies and Their Unmutated Ancestor Antibodies
Source: PLoS Pathog. 2011 Sep 1;7(9):e1002200. doi: 10.1371/journal.ppat.1002200 (PMC3164629; doi:10.1371/journal.ppat.1002200)

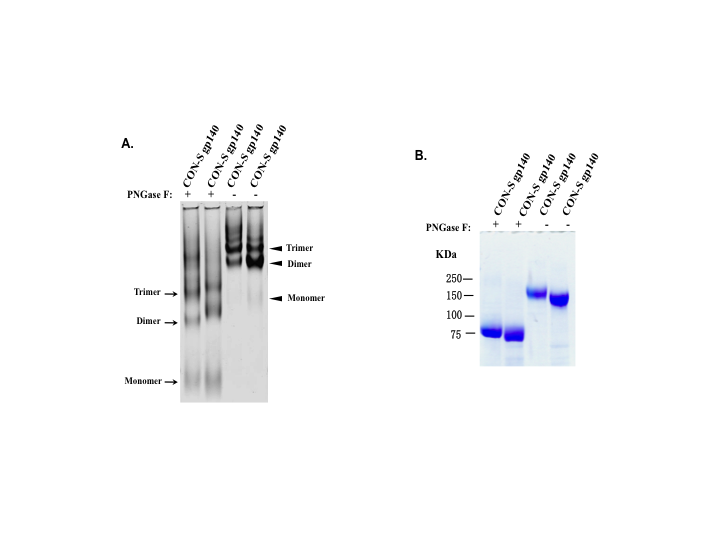

Supplement: Figure S1 — Analysis of deglycosylated JRFL protein in blue native (BN) gel and SDS-PAGE. JRFL and CON-S gp140 Env and JRFL gp140 and the CON-S gp140 Env proteins that were natively deglycosylated using 500U PNGase F/ug Env were fractionated in either blue native gel (Panel S1A) or in 4-12% SDS-PAGE under reducing condition and stained with coommassie blue (Panel S1B). The + and – above the individual lanes indicate that JRFL gp140 or CON-S gp140 were treated (+) or not treated (-) with 500U PNGase F/µg Env. In BN-PAGE gel analysis, deglycosylated JRFL gp140 and CON-S gp140 Envs migrated as monomer, dimer and trimer with decreased molecular weight as results from the removal of glycans compared with the WT glycosylated Envs (panel S1A). Panel B shows that in SDS-PAGE under reducing conditions, treatment with 500U/ug Env PNGase F reduced the molecular weight to approximately 80 kDa for JRFL gp140 CF Env and 75kDa for CON-S gp140 CFI Env. The slightly lower molecular weights of protein bands of CON-S gp140 were due to the deletion of the immunodominant region in the CON-S gp140CFI design compared with JRFL gp140CF protein without deletion of the immunodominant region [29]. Note that in the reduced Coomasie stained SDS-PAGE gels (Panel S1B) there are no bands higher than gp140, indicating compete reduction of the Env dimers and higher MW forms. (TIF) [file ppat.1002200.s001.tif]

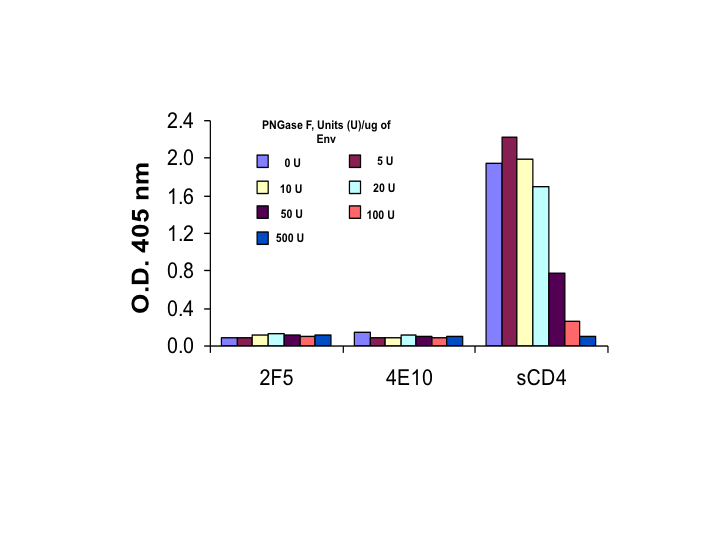

Supplement: Figure S2 — Binding of mAbs 2F5 and 4E10 to deglycosylated Env gp120 proteins. Shown is the evidence of lack of binding in ELISA of mAbs 2F5 and 4E10 to Env gp120 proteins directly coated on ELISA plates after progressive deglycosylation from 0 to 500 U per µg of Env protein (DEG 0-DEG 500). Binding of sCD4 to Env gp120 in this assay setting was maintained on Env gp120 protein up to 100 U of PNGase F. (TIF) [file ppat.1002200.s002.tif]

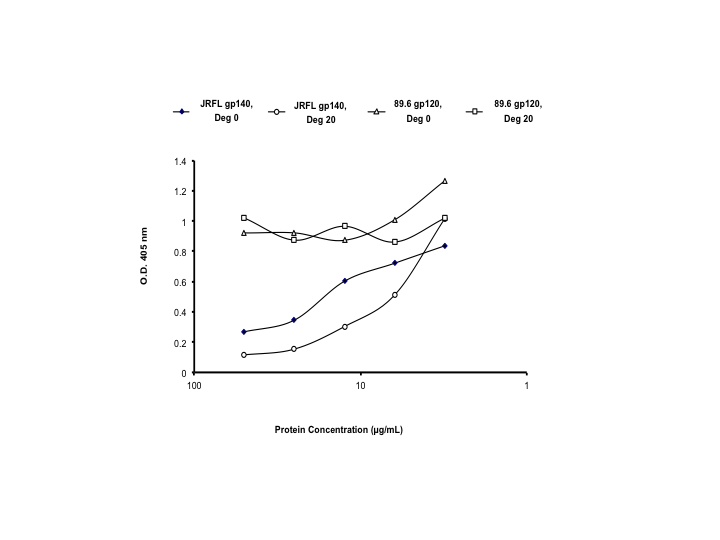

Supplement: Figure S3 — Inhibition of binding of mAb 4E10 to 4E10 binding epitope peptide (P4E10) by WT glycosylated and deglycosylated JRFL 140 protein. MAb 4E10 at 50 µg/ml was first pre-incubated with the indicated concentrations (in x-axis) of HIV-1 Env protein of either WT glycosylated (Deg 0) or moderately deglycosylated (Deg 20, 20 U per µg of protein) JRFL gp140CF or Env gp120, and then assayed for binding to a 4E10 epitope peptide (SLWNWFNITNWLWYIK) by ELISA. Incubation of either WT (Deg 0) or moderately deglycosylated (Deg 20) Env gp120 had little effect on the binding of mAb 4E10 to the 4E10 epitope peptide, while deglycosylated JRFL gp140 protein showed more efficient absorption than did WT glycosylated JRFL Env protein of the binding of mAb 4E10 to the 4E10 epitope peptide. (TIF) [file ppat.1002200.s003.tif]

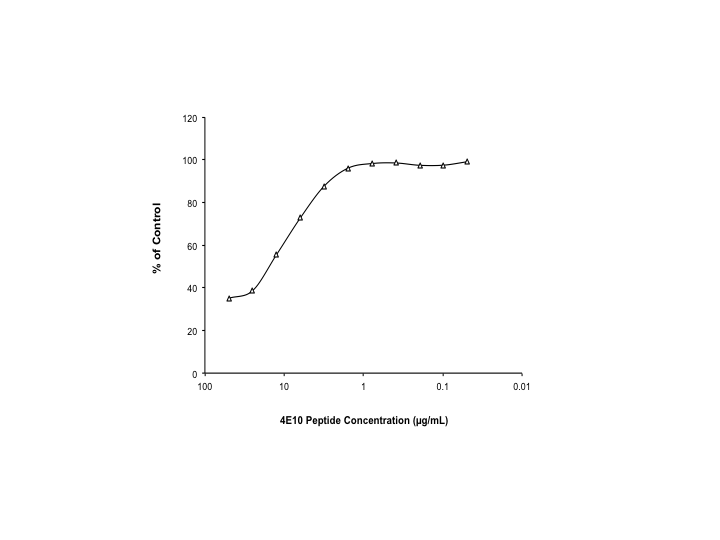

Supplement: Figure S4 — Inhibition of mAb 4E10 binding to deglycosylated JRFL gp140 proteins. MAb 4E10 was pre-incubated with indicated concentrations (x-axis) of 4E10 epitope peptide (SLWNWFNITNWLWYIK) and then assayed in ELISA for binding to deglycosylated (500 U PNGase F) JRFL gp140 Env protein. Percentage of binding (y axis) of absorbed mAb 4E10 to the Env antigen was determined in comparison with the binding of non-absorbed antibody and plotted versus the amount of 4E10 peptide used for absorption. 4E10 peptide (SLWNWFNITNWLWYIK) inhibited the binding of 4E10 mAb to deglycosylated JRFL gp140 in a dose-dependent manner. Data shown are representative of three experiments performed. (TIF) [file ppat.1002200.s004.tif]

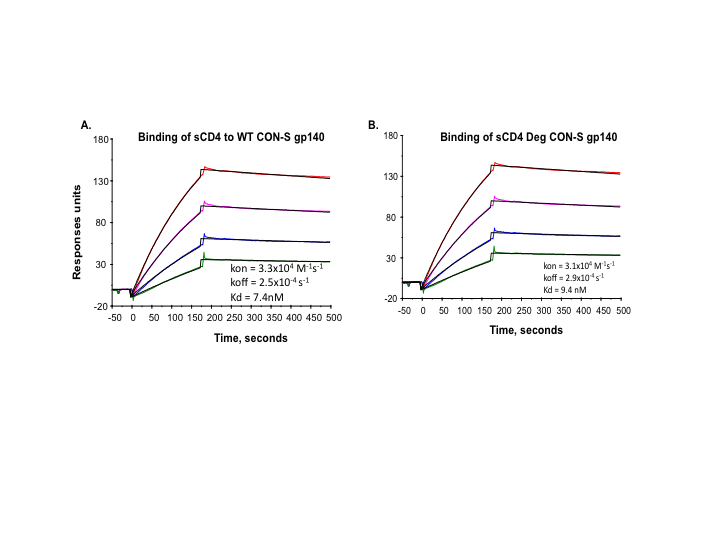

Supplement: Figure S5 — Binding of soluble (s) CD4 to WT glycosylated and deglycosylated CON-S gp140 by SPR. Surface plasmon resonance assays were performed as described in Materials and Methods. Shown are the binding of sCD4 to the WT glycosylated (WT) (Panel S5A) and deglycosylated (Deg) (Panel S5B). sCD4 was covalently immobilized to a CM5 sensor chip (BIAcore), and WT glycosylated and deglycosylated CON-S gp140 injected over each surface (5-20ug/mL and 40-100 µg/mL, respectively). Rate constants and Kd measurements were made using the 1∶1 Langmuir model. The on-rate (Kon), off -rate (Koff) and Kd are indicated in the individual panels. Each analysis was performed at least twice. (TIF) [file ppat.1002200.s005.tif]

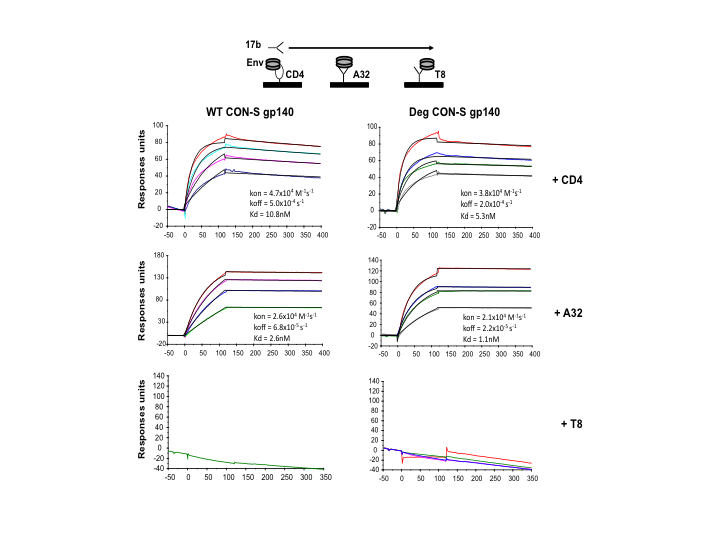

Supplement: Figure S6 — Analysis of antigenic epitopes expressed on WT glycosylated and deglycosylated CON-S gp140 by surface plasmon resonance (SPR). SPR assays were performed as described in Materials and Methods. Shown is the ability of WT glycosylated and deglycosylated CON-S gp140 to bind to mAb 17B (Panel S6B). sCD4 or HIV-1 mAbs T8 and A32 were covalently immobilized to a CM5 sensor chip (BIAcore), and WT glycosylated and deglycosylated CON-S gp140 were injected over each surface to capture 320 – 500 RU of Env proteins. To determine induction of 17b MAb binding to glycosylated and deglycosylated CON-S gp140, Env proteins at equivalent RU amounts were captured on individual flow cells immobilized with sCD4 or mAb A32 or T8. Following stabilization of each of the surfaces, varying concentrations of mAb 17b (25 to 100ug/ml) was injected and allowed to flow over each of the immobilized flow cells as illustrated in the diagram above the SPR profiles in Panel B. The on-rate (kon), off -rate (koff) and Kd are indicated in individual panels. Each analysis was performed at least twice. (TIF) [file ppat.1002200.s006.tif]

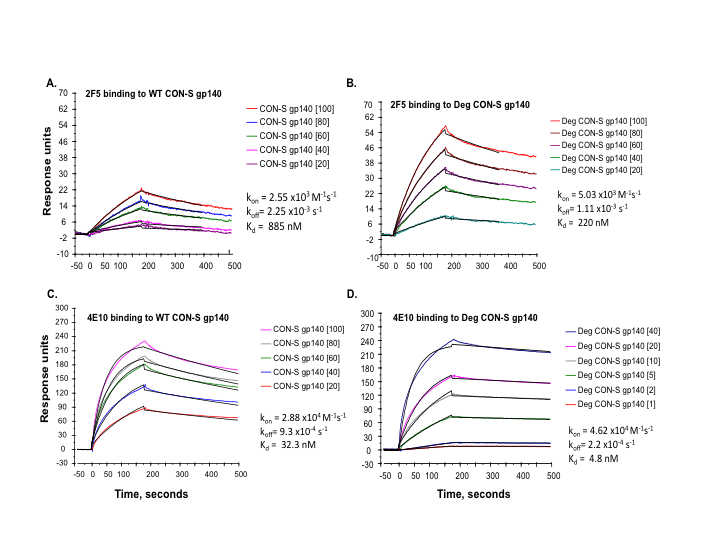

Supplement: Figure S7 — SPR assays of binding of mAbs 4E10 and 2F5 to WT glycosylated and deglycosylated CON-S gp140 Envs. MAbs 2F5 (Panels S7A, S7B) and 4E10 (Panel S7C, S7D) were bound to anti-human Ig Fc immobilized on the CM5 sensor chip. Then varying concentrations (ranging from 20 to 100 µg/ml) of glycosylated (WT) or deglycosylated (Deg) Con S gp140 (20-100 µg/ml or 1 to 40 ug/mL as indicated) proteins were injected over the mAbs on the chip and binding kinetics were recorded. Rate constants and Kd measurements were made using the 1∶1 Langmuir model and data are as indicated. Deglycosylation of CON-S gp140 enhanced 2F5 and 4E10 Kds by ∼4 fold and ∼7-fold, respectively. Each analysis was performed at least twice. (TIF) [file ppat.1002200.s007.tif]

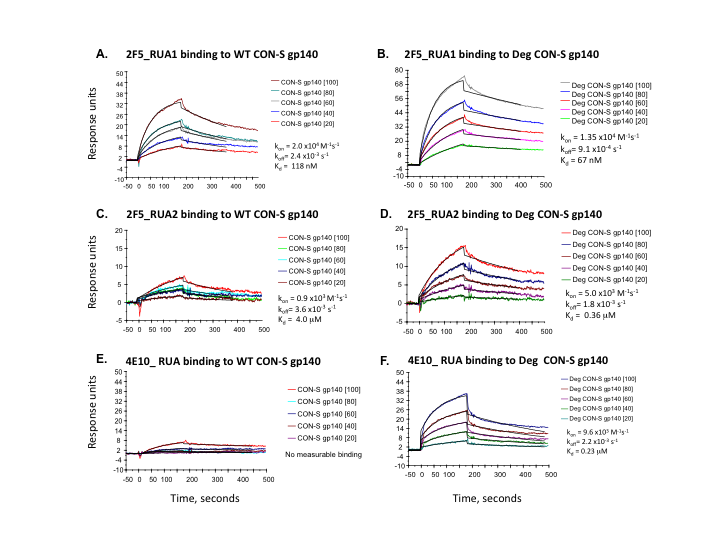

Supplement: Figure S8 — SPR measurements of binding of inferred RUAs of 2F5 (S8A-S8D) and 4E10 (S8E and S8F) to WT glycosylated and deglycosylated CON-S gp140 Envs. Dose ranges (20-100 µg as indicated) of RUA1 (Panels S8A and S8B) and RUA2 (Panels S8C and S8D) inferred from mAb 2F5, and one RUA (Panels S8E and S8F) inferred from mAb 4E10 were tested by SPR for binding to glycosylated (WT) and deglycosylated (Deg). Each antibody was captured on an anti-Ig Fc immobilized chip as described earlier [7], [8], [59]. Rate constants and Kd measurements were made using the 1∶1 Langmuir model. The on-rate (Kon), off -rate (Koff) and Kd are indicated in individual panels. Each analysis was performed at least twice. (TIF) [file ppat.1002200.s008.tiff]
