# Supplementary material for: Envelope Deglycosylation Enhances Antigenicity of HIV-1 gp41 Epitopes for Both Broad Neutralizing Antibodies and Their Unmutated Ancestor Antibodies
Source: PLoS Pathog. 2011 Sep 1;7(9):e1002200. doi: 10.1371/journal.ppat.1002200 (PMC3164629; doi:10.1371/journal.ppat.1002200)
Supplement: Table S1 — Mass spectrometry analysis of deglycosylated JRFL gp140 Env treated with Endo H and Endo F3. (DOC) [file ppat.1002200.s009.doc]

**Table S1.** Mass spectrometry analysis of deglycosylated JRFL gp140 Env treated with Endo H and Endo F3.

| **Env Region** | **CS** | **Experimental Mass** | **Theoretical Mass** | **Mass Error** | **Peptide Sequence** | **Glycan** |
| --- | --- | --- | --- | --- | --- | --- |
| C1 | 3+ | 1493.6996 | 1493.6863 | 9 | AYDTEVHNVWATHACVPTDPNPQEVVLEDVTEHFNMWK |  |
|  | 4+ | 1120.5341 | 1120.5165 | 11 | AYDTEVHNVWATHACVPTDPNPQEVVLEDVTEHFNMWK |  |
|  |  |  |  |  |  |  |
| V1 | 2+ | 928.3910 | 928.3842 | 7 | DVDATNTTNDSEGTMER |  |
|  | 2+ | 928.8830 | 928.8762 | 7 | DVDATDTTNDSEGTMER |  |
|  |  |  |  |  |  |  |
| V1-V2 | 2+ | 657.3136 | 657.3113 | 3 | NCSFDITTSIR |  |
|  | 2+ | 657.8065 | 657.8033 | 5 | DCSFDITTSIR |  |
|  |  |  |  |  |  |  |
| V2 | 2+ | 810.9021 | 810.8968 | 7 | LDVVPIDNDNTSYR |  |
|  |  |  |  |  |  |  |
| C2 | 3+ | 1274.6698 | 1274.6737 | 3 | NVSTVQCTHGIRPVVSTQLLLNGSLAEEEVVIR | [HexNAc] |
|  | 3+ | 1275.0059 | 1275.0017 | 3 | NVSTVQCTHGIRPVVSTQLLLDGSLAEEEVVIR | [HexNAc] |
|  |  |  |  |  |  |  |
|  |  |  |  |  |  |  |
|  | 3+ | 1207.3177 | 1207.3085 | 8 | NVSTVQCTHGIRPVVSTQLLLDGSLAEEEVVIR |  |
|  | 3+ | 1207.6425 | 1207.6366 | 5 | DVSTVQCTHGIRPVVSTQLLLDGSLAEEEVVIR |  |
|  |  |  |  |  |  |  |
|  | 1+ | 1011.4459 | 1011.4378 | 5 | SDDFTNNAK |  |
|  |  |  |  |  |  |  |
| C2-V3 | 2+ | 1004.4557 | 1004.4554 | 0.3 | ESVEINCTRPNDNTR | [HexNAc] |
|  | 2+ | 1105.5041 | 1105.5031 | 0.9 | ESVEINCTRPNNNTR | 2[HexNAc] |
|  |  |  |  |  |  |  |
|  | 2+ | 902.9194 | 902.9157 | 4 | ESVEINCTRPNDNTR |  |
|  | 2+ | 903.4117 | 903.4077 | 4 | ESVEIDCTRPNDNTR |  |
|  | 3+ | 602.6110 | 602.6076 | 6 | ESVEIDCTRPNDNTR |  |
|  |  |  |  |  |  |  |
| V3-C3 | 1+ | 1188.5418 | 1188.5426 | 0.7 | QAHCNISR | [HexNAc] |
|  |  |  |  |  |  |  |
|  | 1+ | 986.4455 | 986.4472 | 2 | QAHCDISR |  |
|  | 2+ | 493.7287 | 493.7272 | 3 | QAHCDISR |  |
|  |  |  |  |  |  |  |
| C3 | 1+ | 777.3800 | 777.3777 | 3 | WDDTLK |  |
|  | 1+ | 979.4717 | 979.4731 | 1 | WNDTLK | [HexNAc] |
|  |  |  |  |  |  |  |
|  | 1+ | 1064.5444 | 1064.5371 | 7 | LREQFEDK |  |
|  | 2+ | 532.7743 | 532.7722 | 4 | LREQFEDK |  |
|  |  |  |  |  |  |  |
|  | 2+ | 903.4216 | 903.4130 | 10 | DKTIVFNHSSGGDPE* | [HexNAc] |
|  | 2+ | 802.3735 | 802.3653 | 10 | DKTIVFDHSSGGDPE* |  |
|  |  |  |  |  |  |  |
| C3-V4 | 2+ | 1397.0803 | 1397.0846 | 3 | FFYCNSTQLFNSTWNNNTE* | [HexNAc]2 |
|  | 2+ | 1397.5882 | 1397.5766 | 8 | FFYCNSTQLFNSTWNDNTE* | [HexNAc]2 |
|  |  |  |  |  |  |  |
| C4 | 2+ | 769.4024 | 769.3981 | 6 | CSSNITGLLLTR | [HexNAc] |
|  | 2+ | 668.3551 | 668.3508 | 6 | CSSDITGLLLTR |  |
|  |  |  |  |  |  |  |
| V5-C5 | 3+ | 698.3196 | 698.3166 | 4 | DGGINEDGTEIFRPGGGDMR |  |
|  |  |  |  |  |  |  |
| TM | 2+ | 924.4708 | 924.4590 | 13 | LICTTAVPWNASWSNK |  |
|  | 2+ | 924.9604 | 924.9510 | 10 | LICTTAVPWNASWSDK |  |
|  |  |  |  |  |  |  |
|  | 2+ | 848.8750 | 848.8660 | 11 | IWNDMTWMEWER |  |
|  |  |  |  |  |  |  |
|  | 2+ | 1337.6392 | 1337.6219 | 13 | EIDNYTSEIYTLIEESQNQQEK | non-glycosylated |

^‡^Note CS indicates charge state. Potential glycosylation sites (N) are in red and deglycosylated sites (D) are in green.

*Peptides resulting from the endoproteinase Glu-C digestion of the envelope protein JR-FL gp140**.**
