# Supplementary material for: Envelope Deglycosylation Enhances Antigenicity of HIV-1 gp41 Epitopes for Both Broad Neutralizing Antibodies and Their Unmutated Ancestor Antibodies
Source: PLoS Pathog. 2011 Sep 1;7(9):e1002200. doi: 10.1371/journal.ppat.1002200 (PMC3164629; doi:10.1371/journal.ppat.1002200)
Supplement: Table S2 — Variable region sequence of mAb 2F5 and 4E10 as well as their inferred reverted and unmutated heavy- and light-chain genes. Shown is the summary of MS data. Each peptide containing a potential glycosylation site was detected as either a glycopeptide, a deglycosylated peptide, or as a nativly nonglycosylated speices. Original regions of HIV-1 Env for the detected individual peptides are indicated. (DOC) [file ppat.1002200.s010.doc]

**Table S2.** Variable region sequence of mAb 2F5 and 4E10 as well as their

inferred reverted and unmutated heavy- and light-chain genes.

>2F5V_H_

AGGATCACGTTAAAGGAATCGGGTCCTCCGCTGGTGAAACCCACACAGACTCTCACGCTGACCTGTTCCTTCTCTGGGTTCTCACTGTCCGATTTTGGAGTGGGTGTAGGCTGGATCCGTCAGCCCCCAGGAAAGGCCCTAGAGTGGCTTGCAATCATTTATTCGGATGATGATAAGCGCTACAGCCCATCGCTGAACACCAGACTCACCATCACCAAGGACACCTCCAAAAATCAAGTTGTCCTTGTCATGACTAGGGTGAGTCCTGTGGACACAGCCACGTATTTCTGTGCACACCGACGGGGGCCAACCACACTCTTTGGAGTGCCGATTGCCCGGGGACCAGTGAACGCGATGGACGTCTGGGGGCAAGGGATCACGGTCACCATCTCCTCA_

>2F5V_K_

GCCCTCCAACTGACCCAGTCTCCGTCCTCCTTGTCTGCATCTGTTGGAGACAGAATCACCATCACTTGTCGGGCAAGTCAGGGCGTTACCAGTGCTTTAGCCTGGTATCGACAGAAGCCAGGAAGTCCTCCTCAACTCCTAATCTATGATGCCTCCTCTTTAGAAAGTGGGGTCCCATCGAGGTTCAGCGGCAGTGGTTCTGGGACGGAGTTCACTCTCACCATCAGCACCCTGCGGCCTGAAGATTTTGCAACTTATTACTGTCAACAATTACATTTTTACCCTCACACTTTCGGCGGAGGGACCAGGGTGGATGTCAGA_

>2F5VH_RUA1

CAGATCACCTTGAAGGAGTCTGGTCCTACGCTGGTGAAACCCACACAGACCCTCACGCTGACCTGCACCTTCTCTGGGTTCTCACTCAGCACTAGTGGAGTGGGTGTGGGCTGGATCCGTCAGCCCCCAGGAAAGGCCCTGGAGTGGCTTGCACTCATTTATTGGAATGATGATAAGCGCTACAGCCCATCTCTGAAGAGCAGGCTCACCATCACCAAGGACACCTCCAAAAACCAGGTGGTCCTTACAATGACCAACATGGACCCTGTGGACACAGCCACATATTACTGTGCACACAGACGGGGGCCAACCACACTTTTTGGAGTGGTTATTGCCCGGGGACCAGTGAACGGTATGGACGTCTGGGGGCAAGGGACCACGGTCACCGTCTCCTCA_

>2F5VH_RUA3

CAGATCACCTTGAAGGAGTCTGGTCCTACGCTGGTGAAACCCACACAGACCCTCACGCTGACCTGCACCTTCTCTGGGTTCTCACTCAGCACTAGTGGAGTGGGTGTGGGCTGGATCCGTCAGCCCCCAGGAAAGGCCCTGGAGTGGCTTGCACTCATTTATTGGGATGATGATAAGCGCTACGGCCCATCTCTGAAGAGCAGGCTCACCATCACCAAGGACACCTCCAAAAACCAGGTGGTCCTTACAATGACCAACATGGACCCTGTGGACACAGCCACATATTACTGTGCACACAGACGGGGGCCAACCACACTTTTTGGAGTGGTTATTGCCCGGGGACCAGTGAACGGTATGGACGTCTGGGGGCAAGGGACCACGGTCACCGTCTCCTCA_

>2F5VK_RUA

GCCATCCAGTTGACCCAGTCTCCATCCTCCCTGTCTGCATCTGTAGGAGACAGAGTCACCATCACTTGCCGGGCAAGTCAGGGCATTAGCAGTGCTTTAGCCTGGTATCAGCAGAAACCAGGGAAAGCTCCTAAGCTCCTGATCTATGATGCCTCCAGTTTGGAAAGTGGGGTCCCATCAAGGTTCAGCGGCAGTGGATCTGGGACAGATTTCACTCTCACCATCAGCAGCCTGCAGCCTGAAGATTTTGCAACTTATTACTGTCAACAGTTTAATAATTACCCTCACACTTTCGGCGGAGGGACCAAGGTGGAGATCAAA_

>4E10V_H_

CAGGTGCAGCTGGTGCAGTCTGGGGCTGAGGTGAAGAGGCCTGGGTCCTCGGTGACGGTCTCCTGCAAGGCTTCTGGAGGCTCCTTCAGCACCTATGCTCTCAGCTGGGTGCGACAGGCCCCTGGACGAGGGCTTGAGTGGATGGGAGGGGTCATCCCTCTCTTAACTATAACAAACTACGCACCGAGGTTCCAGGGCAGAATCACGATTACCGCGGACAGATCCACGAGCACAGCCTACCTGGAGCTGAACAGCCTGAGACCTGAGGACACGGCCGTGTATTACTGTGCGAGAGAAGGGACTACTGGCTGGGGCTGGCTGGGCAAACCCATAGGGGCGTTTGCCCACTGGGGCCAGGGCACCCTGGTCACCGTCTCCTCA_

>4E10V_K_

GAAATTGTGTTGACGCAGTCTCCAGGCACCCAGTCTTTGTCTCCAGGGGAAAGAGCCACCCTCTCCTGCAGGGCCAGTCAGAGTGTTGGCAACAACAAATTAGCCTGGTACCAGCAGAGACCTGGCCAGGCTCCCAGGCTCCTCATCTATGGTGCATCCAGCAGGCCCAGTGGCGTCGCAGACAGGTTCAGTGGCAGTGGGTCTGGGACAGACTTCACTCTCACCATCAGCAGACTGGAGCCTGAAGATTTTGCAGTGTATTACTGTCAGCAGTATGGTCAATCACTCTCAACGTTCGGCCAAGGGACCAAGGTGGAAGTCAAA_

>4E10VH_RUA

CAGGTGCAGCTGGTGCAGTCTGGGGCTGAGGTGAAGAAGCCTGGGTCCTCGGTGAAGGTCTCCTGCAAGGCTTCTGGAGGCACCTTCAGCAGCTATGCTATCAGCTGGGTGCGACAGGCCCCTGGACAAGGGCTTGAGTGGATGGGAGGGATCATCCCTATCTTTGGTACAGCAAACTACGCACAGAAGTTCCAGGGCAGAGTCACGATTACCGCGGACAAATCCACGAGCACAGCCTACATGGAGCTGAGCAGCCTGAGATCTGAGGACACGGCCGTGTATTACTGTGCGAGAGAAGGGACTACTGGCTGGGGCTGGCTGGGCAAACCCATAGGGGCGTTTGACTACTGGGGCCAGGGAACCCTGGTCACCGTCTCCTCA_

>4E10VK_RUA

GAAATTGTGTTGACGCAGTCTCCAGGCACCCTGTCTTTGTCTCCAGGGGAAAGAGCCACCCTCTCCTGCAGGGCCAGTCAGAGTGTTAGCAGCAGCTACTTAGCCTGGTACCAGCAGAAACCTGGCCAGGCTCCCAGGCTCCTCATCTATGGTGCATCCAGCAGGGCCACTGGCATCCCAGACAGGTTCAGTGGCAGTGGGTCTGGGACAGACTTCACTCTCACCATCAGCAGACTGGAGCCTGAAGATTTTGCAGTGTATTACTGTCAGCAGTATGGTAGCTCACCTTGGACGTTCGGCCAAGGGACCAAGGTGGAAATCAAA_
